# Response of broilers subjected to an enteric challenge and fed diets with varying limestone particle sizes and calcium concentrations–part 1: performance, tibia mineralization, and nutrient digestibility^☆^

**DOI:** 10.1016/j.psj.2025.105385

**Published:** 2025-05-31

**Authors:** J.P. Gulizia, R.W. Tabish, J.I. Vargas, J.R. Hernandez, C.T. Simões, E.G. Guzman, S.J. Rochell, R. Hauck, M.A. Bailey, W.A. Dozier, K.W. McCafferty, W.J. Pacheco

**Affiliations:** a Department of Poultry Science, Auburn University, 260 Lem Morrison Dr., Auburn, AL 36849, USA; b Department of Preventive Veterinary, Federal University of Santa Maria, Santa Maria, Rio Grande do Sul, Brazil; c Department of Pathobiology, Auburn University, Auburn, AL 36849, USA; d USDA-ARS Poultry Research Unit, Mississippi State, MS 39762, USA

**Keywords:** limestone particle size, Calcium, *Eimeria*, *Clostridium perfringens*, Broiler

## Abstract

This 35-d experiment evaluated the effects of limestone particle size and calcium concentrations on broiler performance, tibia mineralization, and nutrient digestibility. Two thousand one hundred d-old YPM x 708 male broilers were distributed into 70 floor pens and assigned to 1 of 7 treatments (10 replicates/treatment). This experiment was a 2 × 3 + 1 factorial arrangement, including 2 limestone particle sizes (910 and 200 µm) and 3 calcium concentrations (adequate, reduced, and low), and an unchallenged control. Dietary calcium was reduced in two 0.10 percentage unit steps from adequate concentrations (i.e., breeder recommendations). All factorial treatments were enterically challenged with *Eimeria* spp. and *Clostridium perfringens*. Performance (d 17, 21, 26, and 35), tibia mineralization (d 21 and 35), and nutrient and energy digestibility (d 21 and 35) were measured. The enteric challenge reduced BW and increased feed conversion ratios (**FCR**; contrast, *P* ≤ 0.05). Broilers fed adequate and reduced calcium diets had higher BW, tibia shear strength, and tibia ash on d 35 compared to low calcium diets (main effect, *P* ≤ 0.05). Day 1 to 35 FCR linearly increased as dietary calcium decreased from adequate to low in the group fed 910 µm limestone, whereas in the 200 µm limestone group, only the low calcium and not the reduced calcium led to higher FCR compared with those fed adequate calcium (interaction, *P* ≤ 0.05). Broilers fed reduced and low calcium diets with 910 µm limestone had the highest calcium and phosphorus digestibility on d 21. With 200 µm limestone, broilers fed a low calcium diet had higher mineral digestibility (interaction, *P* ≤ 0.05). Day 35 apparent ileal digestible energy (**AIDE**) increased when broilers consumed an adequate or reduced calcium diet compared to low calcium (main effect, *P* ≤ 0.05). This experiment demonstrated dietary calcium concentrations 0.10 percentage units lower than recommended concentrations can maintain broiler performance, tibia mineralization, and AIDE during an enteric challenge. However, calcium concentration effects depended on limestone particle size.

## Introduction

Antibacterial growth promotors (**AGP**) have been used to improve poultry production efficiency, weight gain, and feed conversion (Miles et al., 2006; Maria Cardinal et al., 2019). However, AGP usage has declined in recent years, which may result in reduced broiler performance and higher incidence of diseases such as coccidiosis and necrotic enteritis (**NE**) (Engster et al., 2002; Cervantes, 2015). Necrotic enteritis is a multifactorial enteric disease caused by *Clostridium perfringens*, particularly after birds have been predisposed by *Eimeria* infection (Emami and Dalloul, 2021). Various nutritional strategies aimed at improving broiler performance and health without AGP have been reviewed to mitigate the severity of these enteric diseases (Dahiya et al., 2006; Ayalew et al., 2022). These nutritional strategies primarily rely on feed additives (e.g., organic acids and probiotics), however, controlling dietary calcium concentrations and particle size of the calcium source could be a viable strategy to mitigate enteric disease severity.

Calcium is an important macro mineral in broiler nutrition, serving as a structural component of bones, intracellular messenger, and cofactor of various enzymes (Proszkowiec-Weglarz and Angel, 2013). While adequate calcium is essential for normal development and physiological processes, broiler diets containing high calcium concentrations or highly soluble calcium sources could negatively impact growth and health, particularly under NE challenge conditions (Paiva et al., 2013; Paiva et al., 2014). Due to the low cost and widespread availability of commonly used calcium sources (David et al., 2023), excessive inclusion of calcium in broiler diets is common, emphasizing the need for stricter control over its concentration (Walk, 2016; Li et al., 2017).

High calcium concentrations may contribute to NE pathogenicity through multiple mechanisms. It can promote the synthesis and activity of NetB and α-toxin produced by *C. perfringens*, which disrupts the intestinal mucosa (Keyburn et al., 2010; Fathima et al., 2022). Furthermore, high calcium concentrations can increase calcium-phytate complex formation (Selle et al., 2009), resulting in undigested nutrients that can serve as substrates for *C. perfringens* (Moran, 2014). Beyond dietary calcium concentration, solubility of the calcium source may also influence NE severity (Paiva et al., 2013).

Limestone is a commonly used calcium source in broiler diets worldwide, but its characteristics vary significantly across regions (Gilani et al., 2022). Variation in limestone characteristics such as particle size can influence its solubility, with smaller particles resulting in higher *in vitro* solubility (de Witt et al., 2006; Kim et al., 2019), but a lower *in vivo* solubility (de Witt et al., 2006). When comparing particulate (402 µm) and pulverized limestone (< 75 µm), Kim et al. (2018) reported that pulverized limestone negatively impacted apparent ileal digestibility (**AID**) of calcium and phosphorus (**P**). Furthermore, Paiva et al. (2013 and 2014) reported a negative impact of higher dietary calcium concentrations and calcium solubility on broiler growth during a naturally occurring clinical NE challenge. Zanu et al. (2020a) expanded on this by examining dietary calcium and phytase concentrations during a subclinical NE challenge, finding that higher dietary calcium combined with the NE challenge reduced CP, carbon, potassium, and zinc digestibility. However, there is still limited data on the response of broilers fed varying limestone particle size and calcium concentrations under subclinical NE conditions. Therefore, the objective of the current experiment was to challenge broilers with *Eimeria* spp. and *C. perfringens* and evaluate the interactive effects of limestone particle size and calcium concentrations on performance, tibia mineralization, and nutrient digestibility.

## Materials and methods

### Animal care

This experiment was conducted at the Auburn University Charles C. Miller Jr. Poultry Research and Education Center. All research procedures were approved by the Auburn University Institutional Biosafety Committee (BUA # 984) and Institutional Animal Care and Use Committee (PRN 2023-5159).

### Bird management

Two thousand one hundred male d-old broiler chicks (YPM x Ross 708; Aviagen North America, Huntsville, AL) were obtained from a commercial hatchery. Upon arrival, chicks were weighed and distributed randomly among 70 floor pens (30 birds/pen, 0.09 m^2^/bird from d 1 to 21; 22 birds/pen, 0.13 m^2^/bird from d 22 to 35) in an environmentally controlled house equipped with exhaust fans, stir fans, forced-air heaters, evaporative-cooling pads, and an electronic controller to manage temperature and ventilation. All floor pens contained new pine shavings with minimal addition of used litter from a previous non-challenged flock. Each pen contained nipple drinkers (5 nipples/pen) and a hanging pan feeder. Feed and water were provided *ad libitum*. The lighting program consisted of 23L:1D from 1 to 7 d and 20L:4D from 8 to 35 d. Environmental temperature was maintained at 33 °C on d 1 and was gradually reduced to 20 °C by 35 d of age.

### Feed formulation, manufacture, and experimental design

All diets were manufactured at the Auburn University Poultry and Animal Nutrition Center and formulated following Aviagen’s recommendations for Ross 708 male broilers (Aviagen, 2022) (Table 1, Table 2). However, calcium concentrations were adjusted in a two-step process, with a 0.10 percentage unit reduction for each growth phase (Starter: Adequate (0.95 %), Reduced (0.85 %), Low (0.75 %); Grower: Adequate (0.85 %), Reduced (0.75 %), Low (0.65 %); Finisher: Adequate (0.75 %), Reduced (0.65 %), Low (0.55 %)). To maintain the balance of other nutrients, sand (inert filler) was added in place of limestone to achieve reduced calcium concentrations. Additionally, amino acid (**AA**) density in the starter feed was formulated at 95.5 % of the recommended primary breeder’s requirements to account for a longer feeding period of the starter phase. Amino acid density in the grower and finisher diets were formulated according to the primary breeder’s recommendations.

**Table 1:** Ingredient composition (% as-fed) of the adequate, reduced, and low calcium (Ca) concentration diets^1^ with varying limestone particle sizes (PS)^2^ fed to YPM x Ross 708 male broilers from 1 to 35 d of age.

Ingredient	Starter, 1 to 17 d			Grower, 18 to 26 d			Finisher, 27 to 35 d		
	Adequate	Reduced	Low	Adequate	Reduced	Low	Adequate	Reduced	Low
Corn	55.46	55.46	55.46	56.82	56.82	56.82	60.42	60.42	60.42
Soybean meal, 48 % CP	39.27	39.27	39.27	36.87	36.87	36.87	33.61	33.61	33.61
DL-methionine	0.37	0.37	0.37	0.36	0.36	0.36	0.33	0.33	0.33
Soy oil	1.83	1.83	1.83	2.84	2.84	2.84	2.95	2.95	2.95
L-lysine	0.21	0.21	0.21	0.18	0.18	0.18	0.14	0.14	0.14
L-threonine	0.13	0.13	0.13	0.11	0.11	0.11	0.08	0.08	0.08
L-valine	0.04	0.04	0.04	0.03	0.03	0.03	0.01	0.01	0.01
Dicalcium phosphate, 18 % P	1.05	1.05	1.05	0.64	0.64	0.64	0.35	0.35	0.35
Limestone (calcium carbonate)	0.90	0.64	0.39	0.92	0.66	0.41	0.87	0.62	0.36
Inert filler^3^ (sand)	0.00	0.26	0.51	0.00	0.26	0.51	0.00	0.26	0.51
Salt	0.50	0.50	0.50	0.50	0.50	0.50	0.50	0.50	0.50
Trace mineral premix^4^	0.10	0.10	0.10	0.10	0.10	0.10	0.10	0.10	0.10
Vitamin premix^5^	0.05	0.05	0.05	0.05	0.05	0.05	0.05	0.05	0.05
Choline chloride	0.06	0.06	0.06	0.05	0.05	0.05	0.05	0.05	0.05
Phytase^6^	0.03	0.03	0.03	0.03	0.03	0.03	0.03	0.03	0.03
Titanium dioxide (TiO_2_)	–	–	–	0.50	0.50	0.50	0.50	0.50	0.50

1 Calcium concentration was a two-step, 0.10 percentage unit reduction from the primary breeder’s requirements for dietary Ca for each of the growth phases (Starter: Adequate (0.95 %), Reduced (0.85 %), Low (0.75 %); Grower: Adequate (0.85 %), Reduced (0.75 %), Low (0.65 %); Finisher: Adequate (0.75 %), Reduced (0.65 %), Low (0.55 %)).

2 Limestone was ground using a 2-pair roller mill (Roskamp Champion Series 900-12, California Pellet Mill Co., Crawfordsville, IN) to achieve a PS of 910 µm (coarse) and 200 µm (fine).

3 Sand was added at the expense of limestone to target reduced calcium concentrations while maintaining all other nutrients.

4 Mineral premix included per kg of diet: Mn (manganese sulfate), 120 mg; Zn (zinc sulfate), 100 mg; Fe (iron sulfate monohydrate), 30 mg; Cu (tri-basic copper chloride), 8 mg; I (ethylenediamine dihydriodide), 1.4 mg; and Se (sodium selenite), 0.3 mg.

5 Vitamin premix included per kg of diet: Vitamin A (Vitamin A acetate), 9,369 IU; Vitamin D (cholecalciferol), 3,307 IU; Vitamin E (DL-alpha tocopherol acetate), 33 IU; menadione (menadione sodium bisulfate complex), 2 mg; Vitamin B12 (cyanocobalamin), 0.02 mg; folacin (folic acid), 1.3 mg; D-pantothenic acid (calcium pantothenate), 15 mg; riboflavin (riboflavin), 11 mg; niacin (niacinamide), 44 mg; thiamine (thiamine mononitrate), 2.8 mg; D-biotin (biotin), 0.09 mg; and pyridoxine (pyridoxine hydrochloride), 3.9 mg.

6 Quantum® Blue 5 G (AB Vista, Marlborough, Wiltshire, UK) provided 1,500 FTU/kg of phytase activity per kg of diet.

**Table 2:** Calculated and analyzed nutrient composition (% as-fed, unless otherwise noted) of the adequate, reduced, and low calcium (Ca) concentration diets^1^ with varying limestone particle sizes (PS)^2^ fed to YPM x Ross 708 male broilers from 1 to 35 d of age.

	Starter, 1 to 17 d			Grower, 18 to 26 d			Finisher, 27 to 35 d		
Calculated nutrients	Adequate	Reduced	Low	Adequate	Reduced	Low	Adequate	Reduced	Low
DM	88.61	88.61	88.61	88.73	88.73	88.73	88.69	88.69	88.69
AMEn, kcal/kg	2,975	2,975	2,975	3,050	3,050	3,050	3,100	3,100	3,100
CP	23.42	23.42	23.42	22.32	22.32	22.32	20.93	20.93	20.93
Ca	0.95	0.85	0.75	0.85	0.75	0.65	0.75	0.65	0.55
Non-phytate phosphorous	0.50	0.50	0.50	0.42	0.42	0.42	0.36	0.36	0.36
Digestible Lys	1.26	1.26	1.26	1.18	1.18	1.18	1.08	1.08	1.08
Digestible Met + Cys	0.95	0.95	0.95	0.92	0.92	0.92	0.86	0.86	0.86
Digestible Met	0.66	0.66	0.66	0.64	0.64	0.64	0.60	0.60	0.60
Digestible Thr	0.84	0.84	0.84	0.79	0.79	0.79	0.72	0.72	0.72
Digestible Val	0.96	0.96	0.96	0.91	0.91	0.91	0.84	0.84	0.84
Analyzed nutrients^3^									
DM^4^	87.99	87.91	87.70	87.71	87.86	87.71	88.22	88.18	88.31
Gross energy^5^, kcal/kg	3,974	3,972	3,944	3,980	4,000	3,971	3,998	3,997	3,994
CP	24.75	22.97	22.02	22.93	22.88	22.27	21.21	21.56	21.04
Ca	0.72	0.63	0.64	0.59	0.52	0.45	0.60	0.46	0.38
Total phosphorus	0.59	0.59	0.59	0.52	0.50	0.49	0.46	0.44	0.45
Phytate-phosphorus^6^	0.25	0.25	0.26	0.24	0.24	0.24	0.23	0.23	0.24
Phytase activity^6^, FTU/kg	1,217	1,365	1,360	1,283	1,375	1,150	1,283	1,405	1,215
Crude fat	4.72	4.76	4.67	5.54	5.61	5.47	5.55	5.41	5.46
Total Lys	–7	–	–	1.48	1.42	1.40	1.36	1.38	1.35
Total Met + Cys	–	–	–	1.06	1.03	1.03	0.96	1.02	1.00
Total Met	–	–	–	0.69	0.66	0.66	0.60	0.65	0.64
Total Thr	–	–	–	0.98	0.92	0.92	0.86	0.88	0.90
Total Val	–	–	–	1.16	1.12	1.10	1.05	1.09	1.05

1 Calcium concentration was a two-step, 0.10 percentage unit reduction from the primary breeder’s requirements for dietary Ca for each of the growth phases (Starter: Adequate (0.95 %), Reduced (0.85 %), Low (0.75 %); Grower: Adequate (0.85 %), Reduced (0.75 %), Low (0.65 %); Finisher: Adequate (0.75 %), Reduced (0.65 %), Low (0.55 %)).

2 Limestone was ground using a 2-pair roller mill (Roskamp Champion Series 900-12, California Pellet Mill Co., Crawfordsville, IN) to achieve a PS of 910 µm (coarse) and 200 µm (fine).

3 Crude protein, calcium, total phosphorus, crude fat, and AA profile analyses were performed by the University of Missouri Agricultural Experiment Station Chemical Laboratories (Columbia, MO); provided are averages of analyzed nutrients for treatments with similar Ca concentrations.

4 Dry matter content was determined by measuring moisture content (AOCS Am 5-04 method, filter bag (ANKOM); analyzed at the University of Missouri Agricultural Experiment Station Chemical Laboratories, Columbia, MO) and subtracting the moisture percentage from 100.

5 Gross energy was analyzed at Auburn University (Auburn, AL); provided are averages of gross energy for treatments with similar Ca concentrations.

6 Phytate-P and phytase activity were analyzed at an external laboratory (AB Vista, Plantation, FL); provided are average values for treatments with similar Ca concentrations.

7 Amino acid content was only analyzed for grower and finisher diets to calculate apparent ileal digestibility.

Broilers were fed a three-phase feeding program consisting of starter (d 1 to 17; crumbles), grower (d 18 to 26; pellets), and finisher diets (d 27 to 35; pellets) and randomly assigned to 1 of 7 treatments with 10 replicates per treatment. This experiment was a 2 × 3 + 1 factorial arrangement including 2 limestone particle sizes (910 and 200 µm) and 3 calcium concentrations (adequate, reduced, and low). The 6 factorially arranged treatments were subjected to an enteric challenge, while the + 1 treatment was an unchallenged control. The unchallenged control birds were fed diets with 200 µm limestone particle size and adequate calcium concentration at each feeding phase. Lastly, all treatments were supplemented with 1,500 FTU/kg of phytase (Quantum Blue 5 G, AB Vista, Marlborough, Wiltshire, UK), which was formulated to provide 0.192 and 0.175 % of calcium and non-phytate P, respectively. Feed samples were sent to an ISO 9001 certified laboratory (AB Vista, Plantation, FL) for phytate-P (NIR analysis of feeds and ingredients using online Feed Quality Service) and phytase activity analysis (QuantiPlate Kits for Quantum Blue® ELISA method (AP181)).

Whole corn was ground with a hammermill (Model 11.5 × 38, Roskamp Champion, Waterloo, IA) equipped with a 4.76 mm screen. Phytase and limestone were premixed with 4.54 kg of ground corn using a countertop mixer (Model A-200, The Hobart Mfg. Co., Troy, OH), prior to their addition to the whole batch of feed. Feed ingredients were blended for 150 s (30 s dry cycle and 120 s wet cycle) using a twin shaft mixer (Model 726, Scott Equipment Co., New Prague, MN) to produce the mash diets. Diets were conditioned at 80 °C for 40 s and pelleted through a 4.4 mm pellet die with an effective thickness of 45 mm (length/diameter ratio = 10.23) using a pellet mill (Model 1112-4, California Pellet Mill Co., Crawfordsville, IN). Pellets were dried and cooled using a counter-flow pellet cooler (Model CC0909, California Pellet Mill Co., Crawfordsville, IN). Starter feed was crumbled in a crumbler with manual roll adjustment (Model 624SS, California Pellet Mill Co., Crawfordsville, IN).

All limestone used for this experiment was received from a single source (Tucker Milling, LLC, Guntersville, AL). Geometric diameter average (**d_gw_**) of particles for the as-received limestone was 2,496 µm. To achieve a particle size of 910 µm (coarse) and 200 µm (fine), as-received limestone was ground using a 2-pair roller mill (Roskamp Champion Series 900-12, California Pellet Mill Co., Crawfordsville, IN). After grinding, limestone particles were separated using a Dura Tap shaker (Advantech, Mentor, OH). Limestone between U.S.A. Standard Test Sieves No. 16 (1,180 µm) and No. 20 (850 µm) were retained for the coarse particle size (910 µm) groups. Whereas limestone between U.S.A. Standard Test Sieves No. 40 (425 µm) and No. 100 (150 µm) were retained for the fine particle size (200 µm) groups. Afterwards, both limestone particle sizes were subjected to particle size analysis using a Dura Tap shaker (Advantech, Mentor, OH) following the ASABE method S319.4 (ASABE, 2008). Duplicate samples of the 910 and 200 µm limestone were sent to an external laboratory (Chemuniqué (Pty) Ltd, Lanseria, South Africa) for nutrient and solubility analysis. Limestone nutrient and solubility analysis was performed using Inductively Coupled Plasma Spectroscopy and methods described by Kim et al. (2019), respectively.

During feed bagging, feed samples were collected at evenly spaced intervals and combined to form a composite sample, which was analyzed in duplicate. Crude protein (combustion analysis (LECO) AOAC Official Method 990.03; AOAC International, 2006), crude fat (by ether extraction, AOAC Official Method 920.39 (A); AOAC International, 2006), minerals (inductively coupled plasma - optical emission spectroscopy (ICP-OES); AOAC Official Method 985.01 (A, B, D) metals and other elements in plants and pet foods; AOAC International, 2006), and complete protein AA profile were analyzed by the University of Missouri Agricultural Experiment Station Chemical Laboratories (Columbia, MO). The AA profile of the feed was analyzed using HPLC (AOAC Official Method 982.30 E (a,b,c), chp 45.3.05; AOAC International, 2006). In addition, tryptophan was analyzed using the AOAC Official Method 988.15, chp. 45.4.04 (AOAC International, 2006). Gross energy (**GE**) of the feed was determined at Auburn University (Auburn, AL) using duplicate 0.75 g samples. Analysis was conducted using an adiabatic oxygen bomb calorimeter (model no. 6400, Parr Instruments, Moline, IA) standardized with benzoic acid.

### Enteric challenge

At 14-d post-hatch, all birds in the challenged groups received an oral gavage with a tenfold dose of a commercially available live attenuated trivalent coccidian vaccine (Advent^ࣨ^; Huvepharma, Inc., Maxton, NC) containing *Eimeria acervulina, Eimeria maxima*, and *Eimeria tenella*. In contrast, the unchallenged groups received a sham gavage of sterile phosphate-buffered saline (**PBS**) as a placebo. On d 17, feed was changed from starter to grower to induce a disturbance in the intestinal microbiota. On d 18, the *Eimeria* challenged groups were orally gavaged with 1 × 10^8^ colony-forming units (**cfu**) of NetB-negative *C. perfringens*. Concurrently, the unchallenged groups were given an equal volume of sterile brain heart infusion broth medium (Becton, Dickinson and Company, Sparks, MD).

### Measurements

Broilers and feed were weighed on d 1 (average chick weight = 46 g), 17, 21, 26, and 35 to determine BW, feed intake (**FI**), and feed conversion ratio (**FCR**). Birds were inspected twice daily and room temperature, bird condition, mortality, and availability of feed and water were monitored during each inspection. The BW of mortalities were used to adjust FCR.

An indigestible marker (0.5 % titanium dioxide, **TiO_2_**; Natracol, ROHA U.S.A., L.L.C., St. Louis, MO) was added to the grower (18 to 26 d of age) and finisher (27 to 35 d of age) feeds to determine apparent ileal nutrient digestibility. On d 21, 8 birds per pen, and on d 35, 6 birds per pen were selected randomly and euthanized by CO_2_ asphyxiation followed by cervical dislocation in accordance with American Veterinary Medical Association Guidelines (AVMA, 2020). Ileal digesta was gently removed starting 2 cm posterior to the Meckel’s diverticulum and ending 2 cm anterior to the ileal-cecal junction to evaluate the digestibility of CP, fat, minerals, energy, and AA. While removing the ileal content, distilled water was used to aid in digesta collection. Ileal contents were pooled within individual pens. Pooled ileal digesta samples were stored on ice, transported to the laboratory, and frozen at -20 °C until analysis. Thereafter, ileal digesta samples were lyophilized in a Virtis Genesis Pilot Lyophilizer (SP Industries, Warminster, PA) and then ground using an electric coffee grinder (Capresso 560.4 Infinity, Montvale, NJ) on the finest setting (< 0.5 mm).

Dried ileal digesta samples were sent to the University of Missouri Agricultural Experiment Station Chemical Laboratories (Columbia, MO) to be analyzed for CP, crude fat, minerals, and AA profile using the methods mentioned previously. Duplicate 0.75 g samples of ileal digesta were analyzed for GE using an adiabatic oxygen bomb calorimeter (model no. 6400, Parr Instruments, Moline, IA) standardized with benzoic acid. Titanium dioxide concentration of feed and ileal digesta was determined through procedures established by Short et al. (1996). Feed (600 mg in quadruplicate) and ileal digesta (200 mg in duplicate) samples were ashed at 580 °C and dissolved in 7.4 M sulfuric acid (J.T. Baker, Avantor, Radnor, PA) at 250 °C. Addition of hydrogen peroxide (30 %; J.T. Baker, Avantor, Radnor, PA) produced a yellow/orange color, with color intensity correlating to TiO_2_ concentration of the sample. Each sample was analyzed in duplicate by measuring 1 mL of the resulting solution using a UV/Vis spectrophotometer (SpectraMax Plus 384 Absorbance Microplate Reader, Molecular Devices, LLC., San Jose, CA) with absorbance measured at 410 nm. Analyzed TiO_2_ concentrations (as-fed basis) for grower feed samples ranged from 0.42 to 0.48 % (average CV and recovery for all grower treatments was 4.08 and 91.12 %, respectively), whereas finisher feed samples ranged from 0.44 to 0.49 % (average CV and recovery for all finisher treatments was 3.49 and 95.25 %, respectively). As for ileal digesta samples, d 21 and 35 CV for TiO_2_ values in all treatments ranged from 2.10 to 7.04 % and 2.38 to 8.17 %, respectively. Apparent ileal digestibility of CP, fat, minerals, energy, and AA were determined using an equation adapted from Stein et al. (2007):

$$\text{AID}\;\%=\left\{\frac{\left[{\left(\frac{\text{Nutrient}}{\text{Ti}O_{2}}\right)}_{\text{diet}}-{\left(\frac{\text{Nutrient}}{\text{Ti}O_{2}}\right)}_{\text{digesta}}\right]}{{\left(\frac{\text{Nutrient}}{\text{Ti}O_{2}}\right)}_{\text{diet}}}\right\}\;x\;100$$

where $\left(\frac{\text{Nutrient}}{\text{Ti}O_{2}}\right)$ represents the ratio of CP, fat, minerals, energy, or AA to TiO_2_ in the diet or ileal digesta. Energy digestibility values obtained from this equation were multiplied by the GE content of feed to determine apparent ileal digestible energy (**AIDE**; DM basis) (Gautier and Rochell, 2020). Digestible nutrient intake (**DNI**) was calculated using an equation adapted from Cowieson et al. (2006):

$$\text{DNI}=\left(CN_{f}\;x\;\text{FI}\right)\;x\;\text{DCN}$$

where DNI indicates the amount of digestible nutrients consumed, measured in g or kcal; CN_f_ represents the nutrient’s concentration in the feed; FI is the feed intake in g; and DCN is the nutrient’s digestibility coefficient, given as a percentage. In the present experiment, DNI was calculated using the grower and finisher feed nutrient concentration, d 17 to 21 and 27 to 35 FI, and the ileal digestibility coefficients determined on d 21 and 35. A detailed account of DNI results were reported (**Supplementary Tables S1-S6** and **Figs. S1-S7**).

In addition, on d 21 and 35, the left tibia from 4 birds/pen were excised to determine tibia weight, shear strength, and ash content. Tibias were placed in sample bags, and frozen at -20 °C before analysis. All tibias were thawed at room temperature and cleaned. Tissue and cartilaginous caps were removed from each tibia. All clean tibias were weighed prior to shear strength assessment. Shear strength of tibias from d 21 were assessed using a TA.XT plus texture analyzer (Stable Microsystems, Surrey, UK), whereas tibias from d 35 were assessed using a TA.XT plus 100C texture analyzer (Stable Microsystems, Surrey, UK) according to the official method (ANSI/ASAE method S459; ASABE, 2017). Due to load capacity limitations, 2 texture analyzers were needed to assess tibia shear strength at different ages. A test speed of 5 mm/sec and a trigger force of 5 g were used for both texture analyzers. The highest peak was recorded in newtons (**N**) as the maximum force to fracture or break the tibia. After breaking all tibias for shear strength determination, a modified bone ash protocol was followed according to Hall et al. (2003). A 6-positioned heating mantle (HM-200-MP6-1000-115, Cole-Parmer, Vernon Hills, IL) was used to house 6 Soxhlet apparatuses (VWR International, Radnor, PA). Tibias were individually packed using cotton cheesecloth (Purewipe Cheesecloth, American Fiber and Finishing Inc., Albemarle, NC) and soaked in 200 proof ethanol (Koptec, Avantor, King of Prussia, PA) for 24 h. Subsequently, tibias were placed in one of the Soxhlet apparatuses and exposed to sequential extractions (24 h in 200 proof pure ethanol followed by 24 h in anhydrous ethyl ether (Supelco, EMD Millipore Corporation, Burlington, MA)). Ethanol was used to remove water and non-polar lipids, whereas anhydrous ether removed polar lipids. Thereafter, tibias were dried at room temperature for a minimum of 24 h and weighed. After weighing, tibias were ashed in a muffle furnace at 580°C. Once ashing was completed, weights were recorded, and tibia ash calculated. Additional samples were collected to evaluate health and physiological measurements, which will be reported in a companion paper (unpublished data).

### Statistical analyses

This experiment was a completely randomized design with data analyzed as a 2 × 3 factorial to evaluate the main effects of limestone particle size and calcium concentration and their interactions during an enteric challenge. There were 2 limestone particle sizes (910 and 200 µm) and 3 calcium concentrations (adequate, reduced, and low). The unchallenged control was excluded from this factorial analysis. However, independent orthogonal contrasts were used to compare the unchallenged control with the challenged treatment, both of which contained 200 µm limestone particle size and adequate calcium concentration.

As a main effect, equally spaced calcium concentrations (Starter: Adequate (0.95 %), Reduced (0.85 %), Low (0.75 %); Grower: Adequate (0.85 %), Reduced (0.75 %), Low (0.65 %); Finisher: Adequate (0.75 %), Reduced (0.65 %), Low (0.55 %)) were evaluated using orthogonal polynomial contrasts to assess linear and quadratic trends in the response. In addition, orthogonal polynomial contrasts were performed for the equally spaced calcium concentrations within each limestone particle size group. Only significant calcium concentration main effects and interactions will be accompanied by orthogonal polynomial contrast *P*-values in the text. All orthogonal polynomial contrast *P*-values are detailed in **Supplementary Tables S7-S14**.

Each treatment was represented by 10 replicate pens with pen considered as the experimental unit. Percentage data were arcsine-transformed before statistical analysis. Using Tukey’s method, outliers were determined when values were 1.5 times the interquartile range below and above the first and third quartile, respectively (Tukey, 1977). All data were analyzed using the GLIMMIX procedure of the SAS software version 9.4 (SAS Institute Inc., Cary, NC). Least square means were compared using Tukey-Kramer with statistical significance considered at *P* ≤ 0.05. For all means, 95 % confidence limits (**CLM**) were determined. If the Tukey-Kramer test was unable to separate the means, then interactions will be described by determining the overlap of 95 % CLM between treatments (Cumming and Fidler, 2009).

## Results and discussion

The roles of calcium in broiler growth and physiology are well-documented and have been extensively reviewed (Adedokun and Adeola, 2013; Proszkowiec-Weglarz and Angel, 2013; David et al., 2023). However, reports highlighting the common occurrence of excess calcium concentrations in broiler diets (Walk, 2016) have generated interest in moving to a digestible calcium system (Walk et al., 2021). Limiting excess dietary calcium during a NE challenge has been an effective approach to control disease related mortality (Paiva et al., 2014). Concomitantly, limestone particle size has been researched for its importance in layers (Hervo et al., 2022), but research is limited in broilers. This is especially important since limestone particle size is highly variable by global region (Gilani et al., 2022). The present experiment evaluated interactive effects of limestone particle size and calcium concentrations on broiler performance, tibia mineralization, and nutrient digestibility during an enteric challenge with *Eimeria* spp. and *C. perfringens*. Further measurements addressing the influence of these interactions on intestinal health and physiology will be discussed in the companion paper (unpublished data).

### Feed and phytase analysis

Analysis of GE, CP, calcium, total P, crude fat, and AA for the adequate, reduced, and low calcium treatments fed during the starter (0.95, 0.85, and 0.75 % calcium), grower (0.85, 0.75, and 0.65 % calcium), and finisher (0.75, 0.65, and 0.55 % calcium) phases are shown in Table 2. All nutrient analyses were averaged between treatments with similar calcium concentrations and different limestone particle sizes. All analyzed dietary calcium concentrations were lower than the calculated values. However, incremental reductions from adequate to low calcium concentrations were achieved for the grower and finisher feed, but not for the starter feed. As detailed in the tibia mineralization section, reduced dietary calcium was confirmed by decreased tibia ash, a sensitive indicator of calcium absorption (Walk et al., 2011; Olgun et al., 2024). On average, starter, grower, and finisher diets contained 0.254 (SD = 0.0049 %; CV = 1.95 %), 0.237 (SD = 0.0045 %; CV = 1.90 %), and 0.233 % (SD = 0.0045 %; CV = 1.94 %) phytate-P, respectively. Dietary phytase was an average of 1,300 (SD = 102 FTU/kg; CV = 7.88 %; Recovery = 86.7 %), 1,271 (SD = 139 FTU/kg; CV = 10.96 %; Recovery = 84.8 %), and 1,299 FTU/kg (SD = 173 FTU/kg; CV = 13.32 %; Recovery = 86.6 %) for the starter, grower, and finisher diets, respectively. Prior to phytase analysis, feed samples were stored for an extended period at 4 °C, which may have resulted in phytase concentrations being below anticipated levels (Singh, 2017).

### Limestone particle size, nutrient, and solubility analysis

**Fig. 1:**
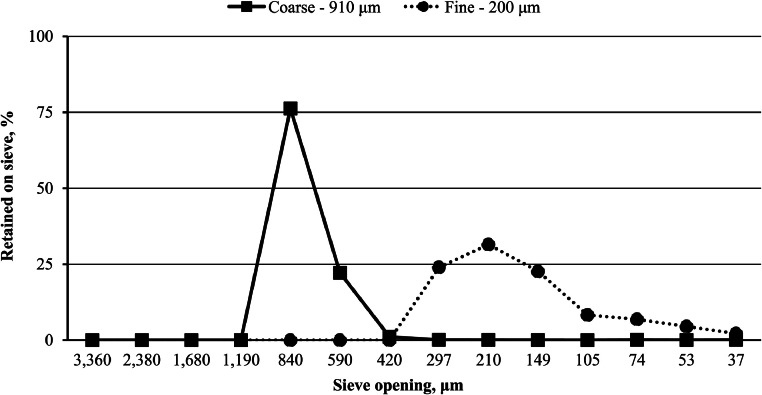
Coarse (910 µm) and fine (200 µm) limestone particle size distributions. Limestone was ground using a 2-pair roller mill (Roskamp Champion Series 900-12, California Pellet Mill Co., Crawfordsville, IN) to achieve distinct particle size distributions.

The limestone particle size distribution is illustrated in Fig. 1. In this experiment, coarse limestone had a d_gw_ of 910 µm with 99.57 % of particles between 1,190 and 420 µm, whereas fine limestone d_gw_ was 200 µm with 100 % of particles between 420 and 37 µm. Limestone nutrient and solubility analyses are shown in Table 3. As expected, analyzed nutrients between the 910 and 200 µm limestone particle size groups were numerically similar. However, solubility of the 200 µm limestone was numerically higher at 5 min (74.77 vs. 42.02 %) and 15 min (95.36 vs. 78.36 %) compared to the 900 µm. By 30 min, the difference in solubility between 910 and 200 µm limestone particle size was only 1.42 % (96.89 vs. 98.31 %). Although minimal, the 200 µm limestone still exhibited slightly higher solubility at this time point. In the present experiment, 2 distinct limestone particle size groups were created with minimal overlap in their particle size distribution. Based on the global survey by Gilani et al. (2022), the 200 µm limestone particle size group closely aligns with the US average. Targeting distinct particle sizes of limestone from the same origin was an attempt to differentiate a solubility effect or interaction with dietary calcium. Increasing calcium solubility can lead to higher concentrations of calcium-phytate complexes (Selle et al., 2009), which may interfere with nutrient utilization (Bedford and Rousseau, 2017). Research indicates that altering limestone particle size influences *in vitro* phytate-P hydrolysis (Manangi and Coon, 2007), *in vitro* solubility, and *in vivo* AID of calcium (Kim et al., 2019). In the present experiment, the d_gw_ range from 200 to 910 µm may not have provided a sufficiently broad particle size distribution to observe consistent effects between the 2 limestone particle size groups. More specifically, pelleting may further reduce ingredient particle size (Bonilla et al., 2022), potentially minimizing the intended differences between limestone particle sizes at 200 and 910 µm. Nevertheless, research by Kim et al. (2018) demonstrated that pulverized limestone (< 75 µm) has higher *in vitro* solubility and is more sensitive to calcium concentration and phytase activity than particulate limestone (402 µm). This also led to *in vivo* effects of particle size on gizzard pH and AID of calcium and P, despite the smaller d_gw_ difference between the 2 limestone particle sizes (327 µm) compared with the present experiment (710 µm).

**Table 3:** Nutrient and solubility analyses of the different limestone particle sizes (PS) fed to YPM x Ross 708 male broilers from 1 to 35 d of age.

	Limestone PS^1^, µm	
Items	910	200
Nutrient analysis^2^		
Moisture, %	0.070	0.030
DM, %	99.93	99.97
Calcium, %	38.20	37.65
Phosphorus, %	0.010	<0.010
Magnesium, %	0.35	0.33
Potassium, %	0.010	0.020
Sodium, %	0.020	0.020
Iron, ppm	391.94	394.93
Manganese, ppm	28.92	29.00
Zinc, ppm	47.06	50.20
Copper, ppm	<0.010	<0.010
Solubility analysis^2^^,^^3^, %		
5 min	42.02	74.77
15 min	78.36	95.36
30 min	96.89	98.31

1 Limestone was ground using a 2-pair roller mill (Roskamp Champion Series 900-12, California Pellet Mill Co., Crawfordsville, IN) to achieve a PS of 910 µm (coarse) and 200 µm (fine).

2 Limestone nutrient and solubility analyses were conducted by Chemuniqué (Pty) Ltd (Lanseria, South Africa).

3 All limestone solubility results were performed in duplicate with the average of both runs reported.

### Broiler performance

Table 4 shows an independent orthogonal contrast between the performance of unchallenged and challenged birds when fed with similar dietary composition. Unchallenged birds had a higher BW (d 21, 26, and 35), higher FI (d 17 to 21, 1 to 21, 18 to 26, and 1 to 26), and lower FCR (d 1 to 21, 18 to 26, 1 to 26, and 1 to 35) compared to enterically challenged broilers (*P* ≤ 0.05). However, mortality was not different between the unchallenged and challenged groups during the entire experiment (*P* > 0.05). In previous work, challenging broilers with *Eimeria* spp. (Wang et al., 2018), *C. perfringens* (Hussein et al., 2020), or both (Zanu et al., 2020a) led to growth impairment. Reduced growth in response to the enteric challenge was partly attributed to lower FI, potentially linked to immune system activation (Dantzer, 2004). The current enteric challenge model successfully reduced broiler performance without affecting mortality, aligning with certain characteristics of a subclinical NE challenge (Palliyeguru and Rose, 2014). However, as will be discussed in the companion paper (unpublished data), limited intestinal lesions were observed in the present experiment, which are an essential criterion for diagnosing subclinical NE (Kaldhusdal and Hofshagen, 1992).

**Table 4:** Independent orthogonal contrast for performance and mortality between unchallenged and challenged YPM x Ross 708 male broilers provided diets varying in limestone particle size (PS) and calcium (Ca) concentration from 1 to 35 d of age^1^.

	200 µm limestone PS^2^ and adequate Ca concentration^3^			
Measurements	Unchallenged	Challenged^4^	CLM^5^	*P*-value
BW, g/bird				
d 17	702	697	± 11	0.517
d 21	1,022	966	± 14	<0.001
d 26	1,567	1,478	± 27	<0.001
d 35	2,632	2,553	± 44	0.013
Feed intake, g/bird				
d 1 to 17	792	790	± 13	0.765
d 17 to 21	433	384	± 8	<0.001
d 1 to 21	1,225	1,180	± 16	<0.001
d 18 to 26	1,157	1,080	± 18	<0.001
d 1 to 26	1,949	1,861	± 28	<0.001
d 27 to 35	1,628	1,653	± 29	0.237
d 1 to 35	3,577	3,530	± 50	0.169
FCR, g:g				
d 1 to 17	1.123	1.131	± 0.008	0.163
d 1 to 21	1.195	1.213	± 0.011	0.023
d 18 to 26	1.345	1.398	± 0.020	<0.001
d 1 to 26	1.234	1.254	± 0.010	0.004
d 27 to 35	1.538	1.528	± 0.016	0.374
d 1 to 35	1.338	1.358	± 0.008	0.002
Mortality, %				
d 1 to 17	1.67	2.33	± 1.63	0.614
d 1 to 21	2.67	3.67	± 2.15	0.587
d 18 to 26	1.67	1.67	± 1.43	0.786
d 1 to 26	3.33	4.00	± 2.32	0.874
d 27 to 35	1.00	0.67	± 1.54	0.863
d 1 to 35	4.33	4.67	± 2.58	0.814

1 Values are least square means of 10 replicate pens, with each pen containing 30 broilers at placement; Statistical significance was considered at *P* ≤ 0.05.

2 Limestone was ground using a 2-pair roller mill (Roskamp Champion Series 900-12, California Pellet Mill Co., Crawfordsville, IN) to achieve a PS of 910 µm (coarse) and 200 µm (fine).

3 Calcium concentration was a two-step, 0.10 percentage unit reduction from the primary breeder’s requirements for dietary Ca for each of the growth phases (Starter: Adequate (0.95 %); Grower: Adequate (0.85 %); Finisher: Adequate (0.75 %)).

4 Broilers were enterically challenged with *Eimeria* spp. and *Clostridium perfringens*.

5 CLM = 95 % confidence limit for the mean.

**Fig. 2:**
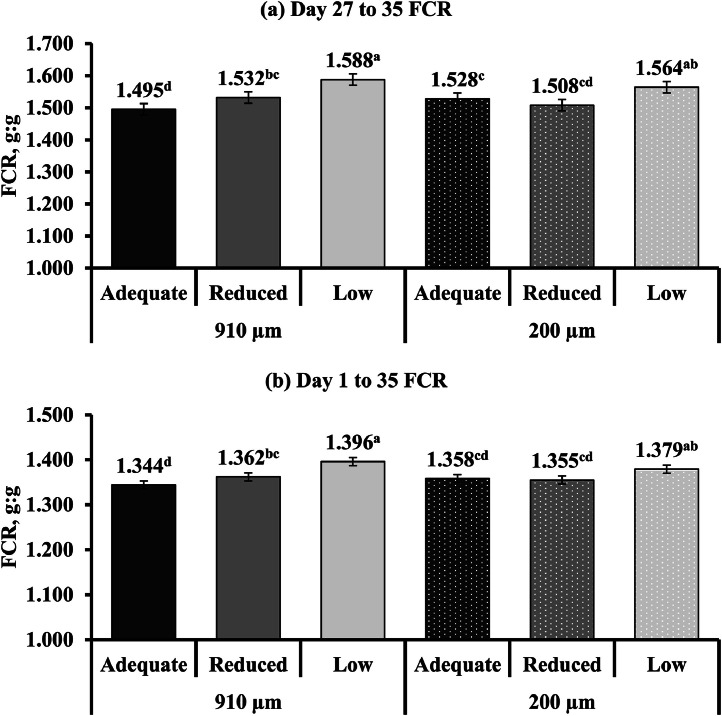
Interaction effects^1^ of varying limestone particle size (910 and 200 µm) and Ca concentrations (adequate, reduced, and low) fed to YPM x Ross 708 male broilers on (**a**) d 27 to 35 (*P* = 0.001; CLM ± 0.018) and (**b**) d 1 to 35 FCR (*P* = 0.001; CLM ± 0.009). ^a-d^Means with different superscripts differ significantly (*P* ≤ 0.05). ^1^Interaction values are least square means of 10 replicate pens, with each pen containing 30 broilers at placement.

The influence of limestone particle size and calcium concentrations on broiler performance is shown in Table 5. Throughout the entire experiment, there were no significant main effects of limestone particle size on broiler performance or mortality (*P* > 0.05). As for calcium concentrations, broilers fed a diet with adequate and reduced calcium had a higher BW and FI compared to broilers fed low calcium throughout the entire experiment (*P* ≤ 0.05). For all BW and FI measurements, the response to calcium concentration was quadratic (*P* ≤ 0.05), except for FI from d 17 to 21, which exhibited a linear response (*P* ≤ 0.05). Broilers fed diets with adequate calcium had a lower FCR compared to those fed diets with reduced and low calcium (1.133 vs. 1.147 and 1.176 g:g) from d 1 to 17 (*P* ≤ 0.05; Quadratic: *P* = 0.017). From d 1 to 21 and d 1 to 26, FCR was lower for broilers fed diets with either adequate or reduced calcium compared to low calcium (*P* ≤ 0.05; Linear: *P* < 0.001). A limestone particle size and calcium concentration interaction was observed for d 27 to 35 and 1 to 35 FCR (*P* ≤ 0.05). Day 27 to 35 FCR for broilers in the 910 µm limestone particle size group exhibited a linear increase (*P* < 0.001) when calcium concentration decreased from adequate to low (Fig. 2 **(a)**). In contrast, the 200 µm limestone particle size group showed a quadratic response (*P* < 0.001), with birds fed adequate and reduced calcium having similar FCR and low calcium leading to a higher FCR. A similar FCR response to calcium concentration in the 910 µm (Linear: *P* < 0.001) and 200 µm (Quadratic: *P* = 0.007) limestone particle size groups was observed from d 1 to 35 (Fig. 2 **(b)**). As for mortality, broilers in the low calcium group had lower d 27 to 35 mortality compared to reduced calcium (*P* ≤ 0.05; Quadratic: *P* = 0.016).

**Table 5:** Performance and mortality of YPM x Ross 708 male broilers provided diets varying in limestone particle size (PS) and calcium (Ca) concentration from 1 to 35 d of age^1^.

	Main effect									Interaction
	Limestone PS^2^, µm (n = 30)				Ca concentration^3^, (n = 20)					Limestone PS x Ca concentration
Measurements	910	200	CLM^4^	*P*-value	Adequate	Reduced	Low	CLM	*P*-value	*P*-value
BW, g/bird										
d 17	675	679	± 6	0.401	693^a^	687^a^	650^b^	± 7	<0.001	0.320
d 21	942	948	± 7	0.259	964^a^	956^a^	914^b^	± 9	<0.001	0.130
d 26	1,445	1,445	± 14	0.955	1,479^a^	1,460^a^	1,397^b^	± 17	<0.001	0.988
d 35	2,515	2,508	± 24	0.603	2,560^a^	2,562^a^	2,411^b^	± 28	<0.001	0.945
Feed intake, g/bird										
d 1 to 17	785	783	± 8	0.711	791^a^	792^a^	769^b^	± 10	0.002	0.958
d 17 to 21	380	380	± 5	0.955	384^a^	385^a^	372^b^	± 6	0.003	0.930
d 1 to 21	1,162	1,166	± 9	0.598	1,179^a^	1,175^a^	1,139^b^	± 11	<0.001	0.499
d 18 to 26	1,065	1,068	± 11	0.718	1,079^a^	1,080^a^	1,040^b^	± 13	<0.001	0.999
d 1 to 26	1,844	1,850	± 16	0.589	1,862^a^	1,869^a^	1,808^b^	± 20	<0.001	0.804
d 27 to 35	1,651	1,630	± 16	0.074	1,652^a^	1,673^a^	1,596^b^	± 20	<0.001	0.230
d 1 to 35	3,497	3,490	± 27	0.728	3,533^a^	3,555^a^	3,393^b^	± 35	<0.001	0.974
FCR, g:g										
d 1 to 17	1.153	1.151	± 0.004	0.479	1.133^c^	1.147^b^	1.176^a^	± 0.005	<0.001	0.127
d 1 to 21	1.230	1.225	± 0.006	0.239	1.214^b^	1.223^b^	1.246^a^	± 0.008	<0.001	0.448
d 18 to 26	1.401	1.399	± 0.011	0.741	1.395	1.397	1.408	± 0.014	0.374	0.653
d 1 to 26	1.270	1.267	± 0.005	0.417	1.256^b^	1.266^b^	1.284^a^	± 0.007	<0.001	0.928
d 27 to 35	1.538	1.534	± 0.009	0.473	1.511	1.520	1.576	± 0.012	<0.001	0.001
d 1 to 35	1.367	1.364	± 0.005	0.275	1.351	1.358	1.388	± 0.006	<0.001	0.001
Mortality, %										
d 1 to 17	1.33	2.44	± 0.95	0.139	1.83	1.83	2.00	± 1.17	0.992	0.887
d 1 to 21	2.33	3.33	± 1.24	0.275	3.17	2.50	2.83	± 1.52	0.671	0.882
d 18 to 26	1.67	1.33	± 0.83	0.386	1.83	1.17	1.50	± 1.01	0.711	0.144
d 1 to 26	3.00	3.78	± 1.35	0.362	3.67	3.00	3.50	± 1.66	0.760	0.580
d 27 to 35	1.78	1.22	± 0.90	0.365	1.17^a^^b^	2.67^a^	0.67^b^	± 1.10	0.046	0.218
d 1 to 35	4.78	5.00	± 1.55	0.649	4.83	5.67	4.17	± 1.89	0.371	0.758

a ^–c^Means within a row with different superscripts differ significantly (*P* ≤ 0.05).

1 Values are least square means of 30 (limestone PS main effect) and 20 (Ca concentration main effect) replicate pens, with each pen containing 30 broilers at placement.

2 Limestone was ground using a 2-pair roller mill (Roskamp Champion Series 900-12, California Pellet Mill Co., Crawfordsville, IN) to achieve a PS of 910 µm (coarse) and 200 µm (fine).

3 Calcium concentration was a two-step, 0.10 percentage unit reduction from the primary breeder’s requirements for dietary Ca for each of the growth phases (Starter: Adequate (0.95 %), Reduced (0.85 %), Low (0.75 %); Grower: Adequate (0.85 %), Reduced (0.75 %), Low (0.65 %); Finisher: Adequate (0.75 %), Reduced (0.65 %), Low (0.55 %)).

4 CLM = 95 % confidence limit for the mean.

Under a mild enteric challenge with *Eimeria* spp. and *C. perfringens*, broiler performance was maintained using adequate and reduced calcium diets. However, decreasing dietary calcium 0.20 percentage units lowered FI, resulting in poor growth. Broilers have exhibited the ability to regulate calcium intake to meet physiological needs (Joshua and Mueller, 1979; Wilkinson et al., 2014), though this ability varies with disease state. Zanu et al. (2020a) observed reduced d 0 to 42 FI when broilers were fed high calcium diets under subclinical NE compared to no challenge. Under a clinical NE challenge, Paiva et al. (2013) reported similar BW gain, FI, and FCR for broilers fed diets with 0.60 or 0.90 % calcium. Similarly, Walk et al. (2012a) observed that broilers not subjected to a disease exhibited comparable performance when fed diets containing 0.64 and 1.03 % calcium. In this experiment, limestone particle size had no effect on BW or FI and limited effects on FCR. Previous research has shown inconsistent broiler performance responses to variations in limestone particle size (Manangi and Coon, 2007; Bradbury et al., 2018; Hu et al., 2020; Majeed et al., 2020). Interactions between calcium concentration and limestone particle size were observed for FCR from d 27 to 35 and 1 to 35. Diets with fine limestone combined with adequate calcium concentrations resulted in higher FCR compared to coarse limestone combined with adequate calcium. This response may be attributed to the higher solubility of finer limestone, which, when combined with higher calcium concentrations, could lower phytate-P hydrolysis (Manangi and Coon, 2007) and increase calcium-phytate complex formation (Selle et al., 2009). Consistent with the present findings, Majeed et al. (2020) and Lee et al. (2021) reported higher FCR when fine limestone was combined with low calcium compared to higher calcium concentrations. Overall, broiler performance was primarily influenced by calcium concentration.

### Tibia mineralization

Table 6 shows an independent orthogonal contrast between the tibia mineralization of unchallenged and challenged birds when fed with similar dietary composition. Tibia weight on d 21, as well as tibia shear strength and tibia ash on d 35, were similar between the unchallenged and enterically challenged broilers (*P* > 0.05). On d 21, tibia shear strength and ash content were higher in broilers from the unchallenged group compared to those challenged with *Eimeria* spp. and *C. perfringens* (*P* ≤ 0.05). Similarly, tibia weight on d 35 was higher in the unchallenged group (*P* ≤ 0.05). Tibia mineralization was reduced when assessed near the administration of the enteric challenge (d 21), which agrees with previous studies (Watson et al., 2005; Akbari Moghaddam Kakhki et al., 2019) and a recent meta-analysis (Shi et al., 2024). Enteric diseases have been associated with impaired bone resorption (Tompkins et al., 2023) and reduced nutrient digestibility (Amerah and Ravindran, 2015; Rochell et al., 2016), both of which contribute to decreased bone mineralization. Similarly, Oikeh et al. (2019) reported that coccidia-infected broilers had reduced plasma calcium and P concentrations, tibia breaking strength, and tibia ash. In the present experiment, tibia ash content was similar between unchallenged and challenged broilers on d 35. This finding is consistent with Zanu et al. (2021), who observed subclinical NE effects on d 16 tibia ash but not on d 29.

**Table 6:** Independent orthogonal contrast for tibia mineralization between unchallenged and challenged YPM x Ross 708 male broilers provided diets varying in limestone particle size (PS) and calcium (Ca) concentration on d 21 and 35^1^.

	200 µm limestone PS^2^ and adequate Ca concentration^3^			
Measurements	Unchallenged	Challenged^4^	CLM^5^	*P*-value
Tibia weight, g				
d 21	4.21	4.21	± 0.13	1.000
d 35	11.77	10.88	± 0.37	0.001
Shear strength, N^6^				
d 21	363	314	± 20	0.001
d 35	490	478	± 22	0.427
Tibia ash, %				
d 21	54.26	53.68	± 0.39	0.040
d 35	52.98	53.16	± 0.59	0.668

1 Values are least square means of 10 replicate pens, with each pen having left tibias collected from 4 broilers on d 21 and 35; Statistical significance was considered at *P* ≤ 0.05.

2 Limestone was ground using a 2-pair roller mill (Roskamp Champion Series 900-12, California Pellet Mill Co., Crawfordsville, IN) to achieve a PS of 910 µm (coarse) and 200 µm (fine).

3 Calcium concentration was a two-step, 0.10 percentage unit reduction from the primary breeder’s requirements for dietary Ca for each of the growth phases (Starter: Adequate (0.95 %), Reduced (0.85 %), Low (0.75 %); Grower: Adequate (0.85 %), Reduced (0.75 %), Low (0.65 %); Finisher: Adequate (0.75 %), Reduced (0.65 %), Low (0.55 %).

4 Broilers were enterically challenged with *Eimeria* spp. and *Clostridium perfringens*.

5 CLM = 95 % confidence limit for the mean.

6 N = Newtons.

The effects of limestone particle size and calcium concentration on tibia mineralization are shown in Table 7. No significant interactions between limestone particle size and calcium concentration were observed for tibia mineralization (*P* > 0.05). Limestone particle size did not affect tibia mineralization on d 21 and 35 (*P* > 0.05). Tibia weight on d 21 (Linear: *P* < 0.001) and d 35 (Quadratic: *P* = 0.019) was highest in broilers fed diets with adequate or reduced calcium compared to broilers fed diets with low calcium (*P* ≤ 0.05). Similarly, broilers fed diets with adequate and reduced calcium had higher d 21 tibia shear strength (Linear: *P* < 0.001) and ash (Linear: *P* < 0.001), as well as d 35 tibia shear strength (Quadratic: *P* = 0.003) and ash (Linear: *P* < 0.001), compared to those fed diets with low calcium (*P* ≤ 0.05). Furthermore, broilers in the adequate calcium group had a higher d 21 tibia shear strength and ash compared to reduced calcium (*P* ≤ 0.05). Under these enteric challenge conditions, reducing dietary calcium concentrations led to lower tibia mineralization, with tibia shear strength and tibia ash displaying similar responses to calcium concentrations. Regardless of a subclinical NE challenge, Zanu et al. (2020b) observed higher tibia breaking strength (d 16 and 29) and tibia ash (d 16 and 29) when dietary calcium was increased by 0.4 percentage units (e.g., 0.5 to 0.9 % in the grower). Similarly, Paiva et al. (2014) reported increased tibia ash on d 35 with higher calcium concentrations (0.90 vs. 0.60 %) during a clinical NE challenge. As observed in previous studies, enteric challenges may influence the tibia mineralization response to dietary calcium. In contrast, Walk et al. (2012b) found no effect of varying calcium concentrations (0.90, 0.75, 0.60, and 0.45 %) on tibia ash in unchallenged broilers. Consistent with the present experiment, limestone particle size has been reported to have no effect on tibia ash (Manangi and Coon, 2007; Majeed et al., 2020). Variability in tibia ash assessment methods (Alkhtib et al., 2023) could explain why tibia ash values here exceed previously established ranges (Alkhtib et al., 2021), though similar values exceeding 50 % have been reported (Walk et al., 2011; Paiva et al., 2014). Bone strength is influenced by multiple factors, including disease and nutrition (Rath et al., 2000). As observed in the present experiment, sufficient calcium concentrations are needed to maintain bone health under this mild enteric challenge using *Eimeria* spp. and *C. perfringens*. Relative to the performance results, reducing calcium by 0.10 percentage units still maintained tibia mineralization at 35 d of age.

**Table 7:** Tibia mineralization of YPM x Ross 708 male broilers provided diets varying in limestone particle size (PS) and calcium (Ca) concentration on d 21 and 35^1^.

	Main effect									Interaction
	Limestone PS^2^, µm (n = 30)				Ca concentration^3^, (n = 20)					Limestone PS x Ca concentration
Measurements	910	200	CLM^4^	*P*-value	Adequate	Reduced	Low	CLM	*P*-value	*P*-value
Tibia weight, g										
d 21	4.06	4.06	± 0.08	0.983	4.21^a^	4.12^a^	3.85^b^	± 0.09	<0.001	0.522
d 35	10.81	10.73	± 0.26	0.634	11.15^a^	11.07^a^	10.09^b^	± 0.32	<0.001	0.159
Shear strength, N^5^										
d 21	291	282	± 12	0.285	316^a^	287^b^	256^c^	± 15	<0.001	0.538
d 35	456	446	± 12	0.216	484^a^	469^a^	399^b^	± 14	<0.001	0.956
Tibia ash, %										
d 21	52.93	53.19	± 0.26	0.132	53.64^a^	53.05^b^	52.49^c^	± 0.32	<0.001	0.574
d 35	52.34	52.50	± 0.35	0.532	52.95^a^	52.73^a^	51.58^b^	± 0.43	<0.001	0.532

### Nutrient digestibility

Table 8 shows an independent orthogonal contrast between the CP, fat, calcium, and P digestibility and AIDE of unchallenged and challenged birds when fed with similar dietary composition. Crude protein, calcium, and P digestibility on d 21 was higher when broilers were subjected to an enteric challenge (*P* ≤ 0.05). However, unchallenged birds had a higher d 21 fat digestibility and AIDE compared to the challenged birds (*P* ≤ 0.05). On d 35, calcium digestibility was higher in the challenged group compared to the unchallenged (*P* ≤ 0.05). However, CP, fat, and P digestibility and AIDE did not differ between the unchallenged and challenged birds (*P* > 0.05). Table 9 summarizes the independent orthogonal contrast for apparent essential and total ileal AA digestibility between the unchallenged and challenged groups. On d 21, the digestibility of all essential and total AA was higher in the enterically challenged broilers compared to the unchallenged group (*P* ≤ 0.05). Unchallenged and challenged broilers had similar d 35 ileal digestibility for all essential and total AA (*P* > 0.05), except for Met digestibility. On d 35, broilers challenged with *Eimeria* spp. and *C. perfringens* had higher Met digestibility compared to the unchallenged broilers (*P* ≤ 0.05). The independent orthogonal contrast for apparent non-essential AA digestibility is presented in Table 10. Broilers challenged with *Eimeria* spp. and *C. perfringens* had higher d 21 digestibility for all non-essential AA compared to the unchallenged broilers (*P* ≤ 0.05). However, by d 35, the digestibility of all non-essential AA was similar between the challenged and unchallenged broilers (*P* > 0.05).

**Table 8:** Independent orthogonal contrast for nutrient digestibility and apparent ileal digestible energy (AIDE) between unchallenged and challenged YPM x Ross 708 male broilers provided diets varying in limestone particle size (PS) and calcium (Ca) concentration on d 21 and 35^1^.

	200 µm limestone PS^2^ and adequate Ca concentration^3^			
Measurements	Unchallenged	Challenged^4^	CLM^5^	*P*-value
CP digestibility, %				
d 21	72.31	78.02	± 1.55	<0.001
d 35	77.60	77.91	± 2.41	0.870
Fat digestibility, %				
d 21	93.15	72.18	± 3.45	<0.001
d 35	92.16	90.90	± 4.56	0.898
Ca digestibility, %				
d 21	45.52	53.88	± 2.85	<0.001
d 35	43.75	60.28	± 5.78	<0.001
P^6^ digestibility, %				
d 21	60.20	77.74	± 1.78	<0.001
d 35	68.24	73.63	± 4.03	0.088
AIDE^7^, kcal/kg				
d 21	3,272	3,036	± 62	<0.001
d 35	3,314	3,332	± 46	0.556

1 Values are least square means of 10 replicate pens, with each pen having ileal digesta collected from 8 and 6 broilers on d 21 and 35, respectively; Statistical significance was considered at *P* ≤ 0.05.

2 Limestone was ground using a 2-pair roller mill (Roskamp Champion Series 900-12, California Pellet Mill Co., Crawfordsville, IN) to achieve a PS of 910 µm (coarse) and 200 µm (fine).

3 Calcium concentration was a two-step, 0.10 percentage unit reduction from the primary breeder’s requirements for dietary Ca for each of the growth phases (Starter: Adequate (0.95 %), Reduced (0.85 %), Low (0.75 %); Grower: Adequate (0.85 %), Reduced (0.75 %), Low (0.65 %); Finisher: Adequate (0.75 %), Reduced (0.65 %), Low (0.55 %).

4 Broilers were enterically challenged with *Eimeria* spp. and *Clostridium perfringens*.

5 CLM = 95 % confidence limit for the mean.

6 P = Phosphorus.

7 On a DM basis.

**Table 9:** Independent orthogonal contrast for apparent essential and total ileal amino acid (AA) digestibility between unchallenged and challenged YPM x Ross 708 male broilers provided diets varying in limestone particle size (PS) and calcium (Ca) concentration on d 21 and 35^1^.

	200 µm limestone PS^2^ and adequate Ca concentration^3^			
Digestibility, %	Unchallenged	Challenged^4^	CLM^5^	*P*-value
Met				
d 21	86.56	89.41	± 1.44	0.004
d 35	86.86	89.54	± 1.73	0.040
Met + Cys				
d 21	76.13	80.32	± 1.61	<0.001
d 35	78.88	80.35	± 2.37	0.371
Lys				
d 21	74.08	80.16	± 1.94	<0.001
d 35	80.20	79.09	± 3.18	0.574
Thr				
d 21	66.33	71.57	± 2.08	<0.001
d 35	72.40	71.46	± 3.23	0.631
Val				
d 21	71.67	76.08	± 2.10	0.003
d 35	76.56	75.51	± 2.81	0.585
Ile				
d 21	71.78	77.01	± 2.08	<0.001
d 35	78.14	76.71	± 2.76	0.428
Arg				
d 21	81.14	85.31	± 1.40	<0.001
d 35	85.72	84.81	± 2.19	0.511
Trp				
d 21	73.88	84.81	± 1.34	<0.001
d 35	80.41	78.28	± 2.87	0.256
Leu				
d 21	74.22	79.10	± 1.97	0.001
d 35	80.01	79.18	± 2.48	0.603
Phe				
d 21	75.37	79.99	± 1.70	<0.001
d 35	80.59	79.62	± 2.47	0.547
His				
d 21	76.48	79.28	± 1.69	0.018
d 35	81.12	79.69	± 2.23	0.331
Total AA				
d 21	74.04	78.73	± 1.71	<0.001
d 35	79.15	78.43	± 2.44	0.630

1 Values are least square means of 10 replicate pens, with each pen having ileal digesta collected from 8 and 6 broilers on d 21 and 35, respectively; Statistical significance was considered at *P* ≤ 0.05.

2 Limestone was ground using a 2-pair roller mill (Roskamp Champion Series 900-12, California Pellet Mill Co., Crawfordsville, IN) to achieve a PS of 910 µm (coarse) and 200 µm (fine).

3 Calcium concentration was a two-step, 0.10 percentage unit reduction from the primary breeder’s requirements for dietary Ca for each of the growth phases (Starter: Adequate (0.95 %), Reduced (0.85 %), Low (0.75 %); Grower: Adequate (0.85 %), Reduced (0.75 %), Low (0.65 %); Finisher: Adequate (0.75 %), Reduced (0.65 %), Low (0.55 %).

4 Broilers were enterically challenged with *Eimeria* spp. and *Clostridium perfringens*.

5 CLM = 95 % confidence limit for the mean.

**Table 10:** Independent orthogonal contrast for apparent non-essential ileal amino acid digestibility between unchallenged and challenged YPM x Ross 708 male broilers provided diets varying in limestone particle size (PS) and calcium (Ca) concentration on d 21 and 35^1^.

	200 µm limestone PS^2^ and adequate Ca concentration^3^			
Digestibility, %	Unchallenged	Challenged^4^	CLM^5^	*P*-value
Ala				
d 21	72.98	76.53	± 2.21	0.017
d 35	77.82	77.50	± 2.72	0.830
Asp				
d 21	71.56	76.20	± 1.66	<0.001
d 35	78.06	76.87	± 2.35	0.424
Cys				
d 21	57.20	63.64	± 2.20	<0.001
d 35	64.09	64.35	± 3.34	0.925
Glu				
d 21	79.59	83.78	± 1.24	<0.001
d 35	84.26	84.25	± 1.94	0.957
Gly				
d 21	67.33	72.08	± 2.07	0.001
d 35	73.75	71.74	± 2.92	0.286
Pro				
d 21	75.55	78.75	± 1.53	0.002
d 35	79.36	79.05	± 1.86	0.771
Ser				
d 21	71.72	76.81	± 1.62	<0.001
d 35	78.84	78.50	± 2.72	0.810
Tyr				
d 21	75.41	79.54	± 1.53	<0.001
d 35	79.60	80.21	± 2.44	0.719

1 Values are least square means of 10 replicate pens, with each pen having ileal digesta collected from 8 and 6 broilers on d 21 and 35, respectively; Statistical significance was considered at *P* ≤ 0.05.

2 Limestone was ground using a 2-pair roller mill (Roskamp Champion Series 900-12, California Pellet Mill Co., Crawfordsville, IN) to achieve a PS of 910 µm (coarse) and 200 µm (fine).

3 Calcium concentration was a two-step, 0.10 percentage unit reduction from the primary breeder’s requirements for dietary Ca for each of the growth phases (Starter: Adequate (0.95 %), Reduced (0.85 %), Low (0.75 %); Grower: Adequate (0.85 %), Reduced (0.75 %), Low (0.65 %); Finisher: Adequate (0.75 %), Reduced (0.65 %), Low (0.55 %).

4 Broilers were enterically challenged with *Eimeria* spp. and *Clostridium perfringens*.

5 CLM = 95 % confidence limit for the mean.

Nutrient digestibility of CP, calcium, P, and AA had an unexpected response when assessed after the co-challenge of *Eimeria* spp. and *C. perfringens*. However, fat digestibility and AIDE were reduced due to the enteric challenge, which is consistent with previous literature (Amerah and Ravindran, 2015; M’Sadeq et al., 2015). Impaired fat digestibility may result from interference with bile acid concentration caused by the enteric disease (Bansal et al., 2021). By the conclusion of the experiment, only calcium and Met digestibility remained higher in the challenged group. Digestible nutrient intake of CP, calcium, and P in challenged birds aligned with this response pattern of nutrient digestibility (**Supplementary Table S1**); however, unchallenged birds had higher total AA intake (**Supplementary Table S2**). Additionally, unchallenged birds had higher FI than challenged birds, which may have contributed to the lower nutrient digestibility by potentially exceeding digestive capacity (Svihus and Hetland, 2001; Svihus, 2014). However, it is understood that *Eimeria* spp. and *C. perfringens* can decrease nutrient digestibility (Williams, 2005; Guo et al., 2014; Rochell et al., 2016), as infections with these pathogens can induce intestinal damage (Wu et al., 2016; Kinstler et al., 2024). Although the response of CP, calcium, P, and AA digestibility is unexpected, Zanu et al. (2020c) also observed increased calcium and P digestibility when broilers were challenged with NE. Other factors could have influenced this response to *Eimeria* spp. and *C. perfringens* including the low virulence of the *C. perfringens* strain in the present experiment (Gharib-Naseri et al., 2019), altered cecal microbiota and short-chain fatty acid production (Stanley et al., 2012; Gharib-Naseri et al., 2019), or protease activity of *C. perfringens* (Blaschek and Solberg, 1978; Allison and Macfarlane, 1989). Furthermore, the observed CP digestibility response in the present experiment was replicated in another experiment from our research group (unpublished data) that utilized the same enteric challenge model. Additional research is needed to understand the reasoning behind the increased CP, calcium, P, and AA digestibility when broilers were enterically challenged with this model.

**Fig. 3:**
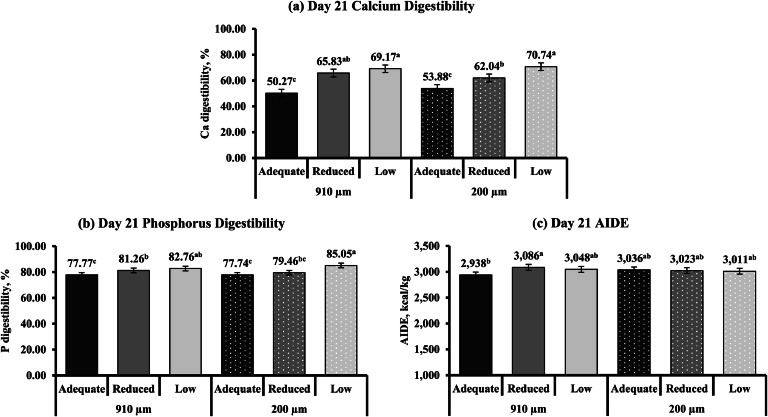
Interaction effects^1^ of varying limestone particle size (910 and 200 µm) and Ca concentrations (adequate, reduced, and low) fed to YPM x Ross 708 male broilers on (**a**) d 21 Ca digestibility (*P* = 0.041; CLM ± 2.93), (**b**) d 21 P digestibility (*P* = 0.047; CLM ± 1.81), and (**c**) d 21 apparent ileal digestible energy (AIDE (on a DM basis); *P* = 0.010; CLM ± 57). ^a-c^Means with different superscripts differ significantly (*P* ≤ 0.05). ^1^Interaction values are least square means of 10 replicate pens, with each pen having ileal digesta collected from 8 broilers on d 21.

The responses of CP, fat, calcium, and P digestibility and AIDE to varying limestone particle size and calcium concentrations on d 21 and 35 are shown in Table 11. Limestone particle size and calcium concentration did not influence CP or fat digestibility (*P* > 0.05). Broilers fed either adequate or reduced calcium diets had a lower d 35 calcium (Linear: *P* = 0.001) and P (Linear: *P* < 0.001) digestibility compared to broilers fed diets with low calcium (*P* ≤ 0.05). On d 35, broilers fed diets with reduced calcium had an intermediate P digestibility. Broilers fed diets with adequate and reduced calcium had higher AIDE on d 35 compared to broilers fed low calcium diets (*P* ≤ 0.05; Linear: *P* < 0.001). Limestone particle size and calcium concentration interactions were observed for d 21 calcium and P digestibility and AIDE (*P* ≤ 0.05). On d 21, calcium digestibility in the 910 µm limestone particle size group showed a quadratic response (*P* = 0.002). Broilers fed an adequate calcium diet had the lowest calcium digestibility, while digestibility increased when broilers consumed a reduced or low calcium diet (Fig. 3 **(a)**). In the 200 µm limestone particle size group, calcium digestibility exhibited a linear increase (*P* < 0.001) as calcium concentration decreased from adequate to low. Day 21 P digestibility linearly increased (*P* < 0.001) when calcium concentration decreased from adequate to low in the 910 µm limestone particle size group (Fig. 3 **(b)**). However, a quadratic response (*P* = 0.041) to calcium concentration was observed in the 200 µm limestone particle size group. Broilers fed adequate and reduced calcium diets had similar P digestibility, whereas digestibility increased when broilers consumed a low calcium diet. Day 21 AIDE exhibited a quadratic response (*P* = 0.008) to calcium concentration in the 910 µm limestone particle size group (Fig. 3 **(c)**). Broilers fed a diet with adequate calcium had lower AIDE compared to broilers fed reduced calcium. Broilers also had similar AIDE when either fed reduced or low calcium diets. In contrast, in the 200 µm limestone particle size group, d 21 AIDE was not different between varying calcium concentrations.

**Table 11:** Nutrient digestibility and apparent ileal digestible energy (AIDE) of YPM x Ross 708 male broilers provided diets varying in limestone particle size (PS) and calcium (Ca) concentration on d 21 and 35^1^.

	Main effect										Interaction
	Limestone PS^2^, µm (n = 30)				Ca concentration^3^, (n = 20)					Limestone PS x Ca concentration	
Measurements	910	200	CLM^4^	*P*-value	Adequate	Reduced	Low	CLM	*P*-value	*P*-value	
CP digestibility, %											
d 21	77.79	77.31	± 0.86	0.426	77.34	77.58	77.73	± 1.07	0.862	0.096	
d 35	78.26	78.66	± 1.27	0.699	78.15	79.14	78.08	± 1.61	0.522	0.471	
Fat digestibility, %											
d 21	72.06	71.68	± 2.38	0.803	70.41	71.88	73.33	± 3.05	0.308	0.111	
d 35	91.85	90.37	± 2.86	0.514	90.79	92.37	90.17	± 3.51	0.740	0.754	
Ca digestibility, %											
d 21	61.76	62.22	± 1.66	0.714	52.08	63.93	69.95	± 2.07	<0.001	0.041	
d 35	67.48	66.23	± 3.08	0.594	62.91^b^	65.31^b^	72.35^a^	± 3.85	0.002	0.353	
P^5^ digestibility, %											
d 21	80.60	80.75	± 0.99	0.720	77.76	80.36	83.90	± 1.25	<0.001	0.047	
d 35	77.99	79.26	± 2.19	0.439	73.69^c^	78.78^b^	83.40^a^	± 2.70	<0.001	0.619	
AIDE^6^, kcal/kg											
d 21	3,024	3,023	± 31	0.979	2,987	3,054	3,030	± 40	0.052	0.010	
d 35	3,339	3,314	± 24	0.137	3,361^a^	3,343^a^	3,277^b^	± 31	<0.001	0.408	

a ^-c^Means within a row with different superscripts differ significantly (*P* ≤ 0.05).

1 Values are least square means of 30 (limestone PS main effect) and 20 (Ca concentration main effect) replicate pens, with each pen having ileal digesta collected from 8 and 6 broilers on d 21 and 35, respectively.

2 Limestone was ground using a 2-pair roller mill (Roskamp Champion Series 900-12, California Pellet Mill Co., Crawfordsville, IN) to achieve a PS of 910 µm (coarse) and 200 µm (fine).

3 Calcium concentration was a two-step, 0.10 percentage unit reduction from the primary breeder’s requirements for dietary Ca for each of the growth phases (Starter: Adequate (0.95 %), Reduced (0.85 %), Low (0.75 %); Grower: Adequate (0.85 %), Reduced (0.75 %), Low (0.65 %); Finisher: Adequate (0.75 %), Reduced (0.65 %), Low (0.55 %).

4 CLM = 95 % confidence limit for the mean.

5 P = Phosphorus.

6 On a DM basis.

**Fig. 4:**
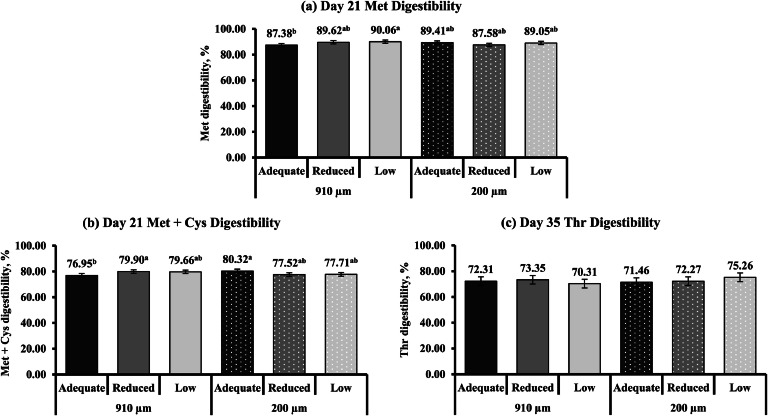
Interaction effects^1^ of varying limestone particle size (910 and 200 µm) and Ca concentrations (adequate, reduced, and low) fed to YPM x Ross 708 male broilers on (**a**) d 21 Met (*P* = 0.007; CLM ± 1.27), (**b**) d 21 Met + Cys digestibility (*P* < 0.001; CLM ± 1.41), and (**c**) d 35 Thr digestibility (*P* = 0.043^†^; CLM ± 3.34). ^a,b^Means with different superscripts differ significantly (*P* ≤ 0.05). ^1^Interaction values are least square means of 10 replicate pens, with each pen having ileal digesta collected from 8 and 6 broilers on d 21 and 35, respectively. ^†^Overall interaction was significantly different; however, Tukey-Kramer was unable to separate the means.

The influence of limestone particle size and calcium concentrations on apparent essential and total ileal AA digestibility is shown in Table 12. Limestone particle size and calcium concentration did not influence apparent essential and total ileal AA digestibility on d 21 or 35 (*P* > 0.05), except for Met (d 21), Met + Cys (d 21), and Thr (d 35) digestibility (*P* ≤ 0.05). Within the 910 µm limestone particle size group, Met digestibility linearly increased (*P* = 0.004) as calcium concentration decreased from adequate to low (Fig. 4 **(a)**). Broilers fed an adequate calcium diet had lower d 21 Met digestibility compared to broilers fed a low calcium diet (*P* ≤ 0.05). In contrast, calcium concentration in the 200 µm limestone particle size group did not influence d 21 Met digestibility. Day 21 Met + Cys digestibility also linearly increased (*P* = 0.009) in the 910 µm limestone particle size group as broilers had lower digestibility when fed an adequate calcium diet compared to a reduced calcium diet (Fig. 4 **(b)**). Calcium concentration did not influence d 21 Met + Cys digestibility in the 200 µm limestone particle size group. Although the Tukey-Kramer mean separation was similar in the 200 µm limestone particle size group, d 21 Met + Cys digestibility exhibited a linear response to calcium concentration (*P* = 0.010). Although an interaction was observed for d 35 Thr digestibility (*P* ≤ 0.05; Fig. 4 **(c)**), the Tukey-Kramer multiple comparison test did not separate the means, and the 95 % CLM overlapped across all treatments.

**Table 12:** Apparent essential and total ileal amino acid (AA) digestibility of YPM x Ross 708 male broilers provided diets varying in limestone particle size (PS) and calcium (Ca) concentration on d 21 and 35^1^.

	Main effect									Interaction
	Limestone PS^2^, µm (n = 30)				Ca concentration^3^, (n = 20)					Limestone PS x Ca concentration
Digestibility, %	910	200	CLM^4^	*P*-value	Adequate	Reduced	Low	CLM	*P*-value	*P*-value
Met										
d 21	89.02	88.68	± 0.73	0.511	88.39	88.60	89.56	± 0.90	0.179	0.007
d 35	89.34	90.17	± 0.94	0.246	89.23	90.03	90.02	± 1.18	0.508	0.861
Met + Cys										
d 21	78.84	78.51	± 0.82	0.598	78.63	78.71	78.68	± 1.00	0.984	<0.001
d 35	79.38	80.25	± 1.24	0.339	80.15	80.77	78.53	± 1.57	0.084	0.883
Lys										
d 21	79.80	80.06	± 1.09	0.710	79.56	79.58	80.65	± 1.34	0.463	0.311
d 35	79.95	80.59	± 1.71	0.641	79.49	80.82	80.50	± 2.15	0.633	0.551
Thr										
d 21	72.26	71.48	± 1.06	0.308	72.22	71.19	72.20	± 1.29	0.483	0.167
d 35	71.99	73.00	± 1.65	0.389	71.89	72.81	72.79	± 2.11	0.742	0.043
Val										
d 21	76.24	75.81	± 1.07	0.584	75.67	75.82	76.59	± 1.31	0.603	0.350
d 35	76.63	77.23	± 1.51	0.606	76.29	77.76	76.75	± 1.91	0.490	0.300
Ile										
d 21	76.63	76.49	± 1.06	0.882	76.34	76.36	76.99	± 1.30	0.761	0.309
d 35	77.65	78.34	± 1.48	0.539	77.31	78.97	77.70	± 1.86	0.373	0.315
Arg										
d 21	85.25	85.34	± 0.75	0.846	85.03	85.23	85.63	± 0.92	0.676	0.703
d 35	85.87	86.18	± 1.16	0.771	85.31	86.74	86.02	± 1.47	0.358	0.370
Trp										
d 21	84.95	85.34	± 0.71	0.430	84.86	85.19	85.38	± 0.90	0.669	0.748
d 35	79.58	79.57	± 1.55	0.950	79.10	79.77	79.87	± 1.95	0.813	0.532
Leu										
d 21	79.11	78.88	± 1.00	0.771	78.45	78.85	79.68	± 1.23	0.385	0.297
d 35	79.47	80.05	± 1.34	0.582	79.42	80.54	79.32	± 1.69	0.467	0.538
Phe										
d 21	79.86	79.82	± 0.86	0.975	79.50	79.60	80.42	± 1.05	0.435	0.352
d 35	80.32	80.76	± 1.32	0.684	80.05	81.43	80.14	± 1.67	0.377	0.367
His										
d 21	78.89	79.01	± 0.91	0.834	78.78	78.99	79.07	± 1.12	0.924	0.461
d 35	80.33	80.68	± 1.19	0.726	80.25	81.44	79.82	± 1.51	0.233	0.297
Total AA										
d 21	78.51	78.50	± 0.87	0.999	78.18	78.38	78.95	± 1.06	0.604	0.342
d 35	79.00	79.47	± 1.30	0.646	78.83	80.10	78.76	± 1.64	0.371	0.370

1 Values are least square means of 30 (limestone PS main effect) and 20 (Ca concentration main effect) replicate pens, with each pen having ileal digesta collected from 8 and 6 broilers on d 21 and 35, respectively.

2 Limestone was ground using a 2-pair roller mill (Roskamp Champion Series 900-12, California Pellet Mill Co., Crawfordsville, IN) to achieve a PS of 910 µm (coarse) and 200 µm (fine).

3 Calcium concentration was a two-step, 0.10 percentage unit reduction from the primary breeder’s requirements for dietary Ca for each of the growth phases (Starter: Adequate (0.95 %), Reduced (0.85 %), Low (0.75 %); Grower: Adequate (0.85 %), Reduced (0.75 %), Low (0.65 %); Finisher: Adequate (0.75 %), Reduced (0.65 %), Low (0.55 %).

4 CLM = 95 % confidence limit for the mean.

**Fig. 5:**
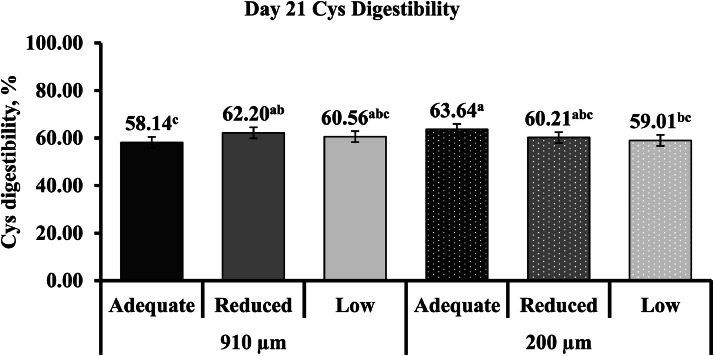
Interaction effects^1^ of varying limestone particle size (910 and 200 µm) and Ca concentrations (adequate, reduced, and low) fed to YPM x Ross 708 male broilers on d 21 Cys digestibility (*P* < 0.001; CLM ± 2.30). ^a-c^Means with different superscripts differ significantly (*P* ≤ 0.05). ^1^Interaction values are least square means of 10 replicate pens, with each pen having ileal digesta collected from 8 broilers on d 21.

Limestone particle size and calcium concentration had minimal influence on apparent non-essential ileal AA digestibility (Table 13). Only d 21 (interaction) and d 35 (calcium concentration main effect) Cys digestibility was influenced by the dietary factors (*P* ≤ 0.05). Day 21 Cys digestibility showed a quadratic increase (*P* = 0.018), with broilers fed a reduced calcium diet and 910 µm limestone particle size exhibiting higher digestibility compared to those fed adequate calcium and the same limestone particle size (Fig. 5). In the 200 µm limestone particle size group, d 21 Cys digestibility linearly decreased (*P* = 0.003) with lower calcium concentrations. Broilers had higher Cys digestibility when fed an adequate calcium diet compared to a low calcium diet. Day 35 Cys digestibility was higher when broilers were fed adequate and reduced calcium diets compared to low calcium (*P* ≤ 0.05; Quadratic: *P* = 0.025).

**Table 13:** Apparent non-essential ileal amino acid digestibility of YPM x Ross 708 male broilers provided diets varying in limestone particle size (PS) and calcium (Ca) concentration on d 21 and 35^1^.

	Main effect									Interaction
	Limestone PS^2^, µm (n = 30)				Ca concentration^3^, (n = 20)					Limestone PS x Ca concentration
Digestibility, %	910	200	CLM^4^	*P*-value	Adequate	Reduced	Low	CLM	*P*-value	*P*-value
Ala										
d 21	76.62	76.47	± 1.12	0.869	75.76	76.42	77.45	± 1.37	0.240	0.286
d 35	78.08	78.38	± 1.44	0.806	77.96	78.86	77.87	± 1.82	0.650	0.573
Asp										
d 21	75.82	75.99	± 0.84	0.749	75.74	75.83	76.14	± 1.03	0.861	0.457
d 35	77.73	78.22	± 1.25	0.611	77.43	79.07	77.44	± 1.58	0.191	0.151
Cys										
d 21	60.30	60.95	± 1.19	0.419	60.89	61.20	59.79	± 1.50	0.339	<0.001
d 35	62.81	62.95	± 1.73	0.925	64.65^a^	64.78^a^	59.20^b^	± 2.18	<0.001	0.705
Glu										
d 21	83.30	83.51	± 0.64	0.597	83.26	83.12	83.83	± 0.79	0.433	0.423
d 35	84.50	85.02	± 1.02	0.517	84.49	85.51	84.28	± 1.29	0.300	0.350
Gly										
d 21	71.55	71.64	± 1.04	0.889	71.28	71.69	71.81	± 1.28	0.828	0.215
d 35	72.74	73.02	± 1.55	0.837	72.49	73.89	72.27	± 1.96	0.389	0.342
Pro										
d 21	78.44	78.30	± 0.78	0.811	78.17	78.45	78.48	± 0.95	0.872	0.240
d 35	78.31	79.09	± 0.97	0.267	78.56	79.65	77.88	± 1.25	0.085	0.662
Ser										
d 21	76.07	76.53	± 0.92	0.458	75.74	76.50	76.66	± 1.16	0.446	0.123
d 35	77.95	78.06	± 1.37	0.968	78.59	78.96	76.46	± 1.76	0.056	0.399
Tyr										
d 21	80.71	80.41	± 0.84	0.626	79.67	80.69	81.32	± 1.06	0.084	0.995
d 35	79.35	80.02	± 1.39	0.517	79.88	80.33	78.84	± 1.71	0.447	0.528

a ^,b^Means within a row with different superscripts differ significantly (*P* ≤ 0.05).

1 Values are least square means of 30 (limestone PS main effect) and 20 (Ca concentration main effect) replicate pens, with each pen having ileal digesta collected from 8 and 6 broilers on d 21 and 35, respectively.

2 Limestone was ground using a 2-pair roller mill (Roskamp Champion Series 900-12, California Pellet Mill Co., Crawfordsville, IN) to achieve a PS of 910 µm (coarse) and 200 µm (fine).

3 Calcium concentration was a two-step, 0.10 percentage unit reduction from the primary breeder’s requirements for dietary Ca for each of the growth phases (Starter: Adequate (0.95 %), Reduced (0.85 %), Low (0.75 %); Grower: Adequate (0.85 %), Reduced (0.75 %), Low (0.65 %); Finisher: Adequate (0.75 %), Reduced (0.65 %), Low (0.55 %).

4 CLM = 95 % confidence limit for the mean.

In this experiment, reducing dietary calcium concentration increased calcium and P digestibility. This suggests that higher dietary calcium concentrations likely increased calcium-phytate complex formation rather than exacerbating enteric disease severity, as CP, fat digestibility, and AIDE did not follow the same trend. In addition, higher calcium concentrations did not negatively affect performance, further supporting the hypothesis that increased calcium-phytate formation, rather than disease severity, influenced calcium and P digestibility. Lower calcium concentrations may reduce calcium-phytate interactions, assisting with phytase activity (Sebastian et al., 1996; Selle et al., 2009), which can improve nutrient digestibility. Also, birds fed lower dietary calcium may have exhibited an adaptive capacity to utilize these minerals by the conclusion of the study (Yan et al., 2005). In contrast to calcium and P digestibility, AIDE was lowered when decreasing dietary calcium by 0.20 percentage units. Adequate calcium concentrations used in this experiment may not have supported high insoluble calcium soap formation, which can influence nutrient utilization (Tancharoenrat and Ravindran, 2014). The effect of dietary calcium on energy utilization is variable, with differing responses reported (Walk et al., 2012b; Tancharoenrat and Ravindran, 2014; Akter et al., 2018). Other than calcium and P, the lack of consistent negative effects from higher dietary calcium concentrations on nutrient digestibility could indicate that the calcium concentrations used in this experiment did not exceed physiological need under enteric challenge conditions. Higher dietary calcium concentrations utilized in other studies have shown adverse effects on nutrient digestibility (Shafey and McDonald, 1991; Mutucumarana et al., 2014; Tancharoenrat and Ravindran, 2014). Nutrient digestibility was minimally affected by dietary calcium concentration and limestone particle size during this mild enteric challenge. However, interactions between calcium and limestone particle size were observed for calcium, P, Met, Met + Cys, Thr, Cys digestibility, and AIDE. As previously discussed, altering limestone particle size can affect its solubility, which can influence nutrient digestibility in response to dietary calcium. Calcium and P digestibility followed a similar response regardless of particles size, with the lowest digestibility at adequate calcium concentrations and the highest at the lowest calcium concentrations. Interestingly, AIDE and the digestibility of Met, Met + Cys, and Thr were all similar within the finest limestone particle size group, regardless of calcium concentration. This suggests that coarser limestone may increase variability in nutrient digestibility. Researchers have reported the effects of limestone particle size on nutrient digestibility (Bradbury et al., 2018; Kim et al., 2018; Kim et al., 2019; Majeed et al., 2020; Li et al., 2021). Majeed et al. (2020) reported that fine limestone particle size (190 µm) increased AA digestibility compared with coarse particle size (900 µm). These responses are likely related to limestone solubility, though they depend on additional dietary factors, including calcium and phytase concentrations (Bradbury et al., 2018; Kim et al., 2018; Kim et al., 2019; Majeed et al., 2020; Li et al., 2021).

In conclusion, the *Eimeria* spp. and *C. perfringens* challenge reduced BW and increased FCR, highlighting its negative impact on performance. Under this enteric challenge model, broiler performance, tibia mineralization, and nutrient digestibility were primarily influenced by dietary calcium concentrations. Reducing dietary calcium by 0.10 percentage units from recommended concentrations maintained performance and tibia mineralization, while a 0.20 percentage unit reduction impaired growth. Thus, lowering calcium beyond physiological needs impacted broiler performance under this mild enteric challenge. More specifically, performance responses suggest that dietary calcium could be reduced to approximately 0.63, 0.52, and 0.46 % analyzed calcium for the 1 to 17, 18 to 26, and 27 to 35 d phases, respectively, in diets supplemented with 1,500 FTU/kg of phytase. Limestone particle size had minimal effects in general, but did interact with calcium concentration to influence FCR and nutrient digestibility. Targeting optimal calcium concentrations and considering limestone particle size when formulating broiler diets may enhance broiler growth and nutrient digestibility under an enteric challenge with *Eimeria* spp. and *C. perfringens*. The severity and type of enteric disease may further modulate these interactions.

## Funding source

This research was funded impart by a USDA-ARS agreement and by the Alabama Agricultural Experiment Station.

## Declaration of competing interest

The authors declare that they have no known competing financial interests or personal relationships that could have appeared to influence the work reported in this paper.

## Appendix Supplementary materials

Image, application 1
