# Supplementary material for: Response of broilers subjected to an enteric challenge and fed diets with varying limestone particle sizes and calcium concentrations–part 1: performance, tibia mineralization, and nutrient digestibility
Source: Poult Sci. 2025 May 31;104(8):105385. doi: 10.1016/j.psj.2025.105385 (PMC12169744; doi:10.1016/j.psj.2025.105385)
Supplement: Supplementary file 1 [file mmc1.docx]

| **Table S1**. Independent orthogonal contrast for digestible nutrient intake (units/bird; DM basis) between unchallenged and challenged YPM x Ross 708 male broilers provided diets varying in limestone particle size (PS) and calcium (Ca) concentration between d 17 to 21 and 27 to 35^1^. | | | | |
| --- | --- | --- | --- | --- |
|  | 200 µm limestone PS^2^ and adequate Ca concentration^3^ | | | |
|  |  |  |  |  |
| Digestible nutrient intake | Unchallenged | Challenged^4^ | CLM^5^ | *P*-value |
| CP, g |  |  |  |  |
| d 17 to 21 | 77.42 | 81.17 | ± 2.20 | 0.019 |
| d 27 to 35 | 305 | 311 | ± 11 | 0.471 |
| Fat, g |  |  |  |  |
| d 17 to 21 | 25.79 | 17.11 | ± 0.94 | <0.001 |
| d 27 to 35 | 87.08 | 91.89 | ± 4.81 | 0.163 |
| Ca, g |  |  |  |  |
| d 17 to 21 | 1.38 | 1.51 | ± 0.08 | 0.021 |
| d 27 to 35 | 4.10 | 6.76 | ± 0.50 | <0.001 |
| P^6^, g |  |  |  |  |
| d 17 to 21 | 1.50 | 1.78 | ± 0.05 | <0.001 |
| d 27 to 35 | 5.63 | 6.50 | ± 0.34 | 0.001 |
| Calorie, kcal |  |  |  |  |
| d 17 to 21 | 1,402 | 1,165 | ± 34 | <0.001 |
| d 27 to 35 | 5,366 | 5,490 | ± 151 | 0.224 |
| ^1^Values are least square means of 10 replicate pens; Statistical significance was considered at *P* ≤ 0.05.  ^2^Limestone was ground using a 2-pair roller mill (Roskamp Champion Series 900-12, California Pellet Mill Co., Crawfordsville, IN) to achieve a PS of 910 µm (coarse) and 200 µm (fine).  ^3^Calcium concentration was a two-step, 0.10 percentage unit reduction from the primary breeder’s requirements for dietary Ca for each of the growth phases (Starter: Adequate (0.95 %), Reduced (0.85 %), Low (0.75 %); Grower: Adequate (0.85 %), Reduced (0.75 %), Low (0.65 %); Finisher: Adequate (0.75 %), Reduced (0.65 %), Low (0.55 %).  ^4^Broilers were enterically challenged with *Eimeria* spp. and *Clostridium perfringens*.  ^5^CLM = 95% confidence limit for the mean.  ^6^P = Phosphorus. | | | | |

| **Table S2**. Independent orthogonal contrast for digestible essential and total amino acid intake (units/bird; DM basis) between unchallenged and challenged YPM x Ross 708 male broilers provided diets varying in limestone particle size (PS) and calcium (Ca) concentration between d 17 to 21 and 27 to 35^1^. | | | | |
| --- | --- | --- | --- | --- |
|  | 200 µm limestone PS^2^ and adequate Ca concentration^3^ | | | |
|  |  |  |  |  |
| Digestible nutrient intake | Unchallenged | Challenged^4^ | CLM^5^ | *P*-value |
| Met, g |  |  |  |  |
| d 17 to 21 | 2.97 | 2.84 | ± 0.07 | 0.004 |
| d 27 to 35 | 9.43 | 10.62 | ± 0.30 | <0.001 |
| Met + Cys, g |  |  |  |  |
| d 17 to 21 | 4.05 | 3.94 | ± 0.11 | 0.172 |
| d 27 to 35 | 13.59 | 15.02 | ± 0.50 | <0.001 |
| Lys, g |  |  |  |  |
| d 17 to 21 | 5.48 | 5.27 | ± 0.16 | 0.064 |
| d 27 to 35 | 20.87 | 19.60 | ± 0.83 | 0.030 |
| Thr, g |  |  |  |  |
| d 17 to 21 | 3.14 | 3.00 | ± 0.12 | 0.080 |
| d 27 to 35 | 11.80 | 11.33 | ± 0.53 | 0.184 |
| Val, g |  |  |  |  |
| d 17 to 21 | 4.20 | 3.85 | ± 0.14 | 0.001 |
| d 27 to 35 | 15.17 | 14.22 | ± 0.61 | 0.027 |
| Ile, g |  |  |  |  |
| d 17 to 21 | 3.64 | 3.48 | ± 0.12 | 0.058 |
| d 27 to 35 | 13.71 | 12.88 | ± 0.53 | 0.026 |
| Arg, g |  |  |  |  |
| d 17 to 21 | 5.95 | 5.57 | ± 0.14 | <0.001 |
| d 27 to 35 | 22.70 | 21.61 | ± 0.70 | 0.026 |
| Trp, g |  |  |  |  |
| d 17 to 21 | 0.96 | 1.07 | ± 0.03 | <0.001 |
| d 27 to 35 | 3.88 | 3.72 | ± 0.14 | 0.104 |
| Leu, g |  |  |  |  |
| d 17 to 21 | 7.05 | 6.69 | ± 0.22 | 0.020 |
| d 27 to 35 | 26.62 | 25.71 | ± 0.96 | 0.176 |
| Phe, g |  |  |  |  |
| d 17 to 21 | 4.24 | 3.99 | ± 0.12 | 0.006 |
| d 27 to 35 | 15.87 | 15.42 | ± 0.57 | 0.270 |
| His, g |  |  |  |  |
| d 17 to 21 | 2.30 | 2.10 | ± 0.07 | <0.001 |
| d 27 to 35 | 8.42 | 8.01 | ± 0.30 | 0.043 |
| Total AA, g |  |  |  |  |
| d 17 to 21 | 84.05 | 79.55 | ± 2.44 | 0.011 |
| d 27 to 35 | 316 | 303 | ± 11 | 0.098 |
| ^1^Values are least square means of 10 replicate pens; Statistical significance was considered at *P* ≤ 0.05.  ^2^Limestone was ground using a 2-pair roller mill (Roskamp Champion Series 900-12, California Pellet Mill Co., Crawfordsville, IN) to achieve a PS of 910 µm (coarse) and 200 µm (fine).  ^3^Calcium concentration was a two-step, 0.10 percentage unit reduction from the primary breeder’s requirements for dietary Ca for each of the growth phases (Starter: Adequate (0.95 %), Reduced (0.85 %), Low (0.75 %); Grower: Adequate (0.85 %), Reduced (0.75 %), Low (0.65 %); Finisher: Adequate (0.75 %), Reduced (0.65 %), Low (0.55 %).  ^4^Broilers were enterically challenged with *Eimeria* spp. and *Clostridium perfringens*.  ^5^CLM = 95% confidence limit for the mean. | | | | |

| **Table S3**. Independent orthogonal contrast for digestible non-essential amino acid intake (units/bird; DM basis) between unchallenged and challenged YPM x Ross 708 male broilers provided diets varying in limestone particle size (PS) and calcium (Ca) concentration between d 17 to 21 and 27 to 35^1^. | | | | |  |
| --- | --- | --- | --- | --- | --- |
|  | 200 µm limestone PS^2^ and adequate Ca concentration^3^ | | | | |
|  |  |  |  |  |  |
| Digestible nutrient intake | Unchallenged | Challenged^4^ | CLM^5^ | *P*-value | |
| Ala, g |  |  |  |  | |
| d 17 to 21 | 4.13 | 3.77 | ± 0.15 | 0.001 | |
| d 27 to 35 | 15.43 | 15.01 | ± 0.59 | 0.318 | |
| Asp, g |  |  |  |  | |
| d 17 to 21 | 8.29 | 7.86 | ± 0.24 | 0.014 | |
| d 27 to 35 | 32.03 | 30.45 | ± 1.12 | 0.041 | |
| Cys, g |  |  |  |  | |
| d 17 to 21 | 1.09 | 1.10 | ± 0.04 | 0.751 | |
| d 27 to 35 | 4.16 | 4.40 | ± 0.24 | 0.139 | |
| Glu, g |  |  |  |  | |
| d 17 to 21 | 16.35 | 15.28 | ± 0.37 | <0.001 | |
| d 27 to 35 | 60.94 | 59.30 | ± 1.84 | 0.191 | |
| Gly, g |  |  |  |  | |
| d 17 to 21 | 3.18 | 3.02 | ± 0.11 | 0.040 | |
| d 27 to 35 | 12.37 | 11.48 | ± 0.51 | 0.014 | |
| Pro, g |  |  |  |  | |
| d 17 to 21 | 4.90 | 4.45 | ± 0.15 | <0.001 | |
| d 27 to 35 | 17.71 | 17.44 | ± 0.54 | 0.481 | |
| Ser, g |  |  |  |  | |
| d 17 to 21 | 3.42 | 3.35 | ± 0.10 | 0.302 | |
| d 27 to 35 | 13.73 | 13.58 | ± 0.48 | 0.645 | |
| Tyr, g |  |  |  |  | |
| d 17 to 21 | 2.72 | 2.49 | ± 0.08 | <0.001 | |
| d 27 to 35 | 10.47 | 10.17 | ± 0.39 | 0.286 | |
| ^1^Values are least square means of 10 replicate pens; Statistical significance was considered at *P* ≤ 0.05.  ^2^Limestone was ground using a 2-pair roller mill (Roskamp Champion Series 900-12, California Pellet Mill Co., Crawfordsville, IN) to achieve a PS of 910 µm (coarse) and 200 µm (fine).  ^3^Calcium concentration was a two-step, 0.10 percentage unit reduction from the primary breeder’s requirements for dietary Ca for each of the growth phases (Starter: Adequate (0.95 %), Reduced (0.85 %), Low (0.75 %); Grower: Adequate (0.85 %), Reduced (0.75 %), Low (0.65 %); Finisher: Adequate (0.75 %), Reduced (0.65 %), Low (0.55 %).  ^4^Broilers were enterically challenged with *Eimeria* spp. and *Clostridium perfringens*.  ^5^CLM = 95% confidence limit for the mean. | | | | |  |

| **Table S4**. Digestible nutrient intake (units/bird; DM basis) of YPM x Ross 708 male broilers provided diets varying in limestone particle size (PS) and calcium (Ca) concentration between d 17 to 21 and 27 to 35^1^. | | | | | | | | | | | | |
| --- | --- | --- | --- | --- | --- | --- | --- | --- | --- | --- | --- | --- |
|  | Main effect | | | | | | | | | |  | Interaction |
|  | Limestone PS^2^, µm (n = 30) | | | |  | Ca concentration^3^, (n = 20) | | | | |  | Limestone PS x  Ca concentration |
| Digestible nutrient intake | 910 | 200 | CLM^4^ | *P*-value |  | Adequate | Reduced | Low | CLM | *P*-value |  | *P*-value |
| CP, g |  |  |  |  |  |  |  |  |  |  |  |  |
| d 17 to 21 | 76.61 | 77.46 | ± 1.36 | 0.371 |  | 79.45 | 78.07 | 73.59 | ± 1.65 | <0.001 |  | 0.031 |
| d 27 to 35 | 310 | 314 | ± 6 | 0.299 |  | 310^b^ | 326^a^ | 300^b^ | ± 8 | <0.001 |  | 0.114 |
| Fat, g |  |  |  |  |  |  |  |  |  |  |  |  |
| d 17 to 21 | 17.31 | 17.13 | ± 0.61 | 0.670 |  | 16.93 | 17.76 | 16.98 | ± 0.76 | 0.218 |  | 0.615 |
| d 27 to 35 | 92.92 | 95.00 | ± 3.16 | 0.344 |  | 96.41 | 96.31 | 89.16 | ± 3.97 | 0.011 |  | 0.001 |
| Ca, g |  |  |  |  |  |  |  |  |  |  |  |  |
| d 17 to 21 | 1.38 | 1.37 | ± 0.04 | 0.901 |  | 1.36 | 1.43 | 1.33 | ± 0.06 | 0.043 |  | <0.001 |
| d 27 to 35 | 6.37 | 5.76 | ± 0.27 | 0.001 |  | 7.47 | 5.74 | 4.99 | ± 0.34 | <0.001 |  | 0.005 |
| P^5^, g |  |  |  |  |  |  |  |  |  |  |  |  |
| d 17 to 21 | 1.71^b^ | 1.80^a^ | ± 0.03 | <0.001 |  | 1.76 | 1.76 | 1.74 | ± 0.04 | 0.695 |  | 0.120 |
| d 27 to 35 | 6.42 | 6.67 | ± 0.19 | 0.072 |  | 6.25^b^ | 6.59^ab^ | 6.81^a^ | ± 0.24 | 0.005 |  | 0.081 |
| Calorie, kcal |  |  |  |  |  |  |  |  |  |  |  |  |
| d 17 to 21 | 1,154 | 1,152 | ± 21 | 0.874 |  | 1,145^ab^ | 1,186^a^ | 1,129^b^ | ± 27 | 0.010 |  | 0.126 |
| d 27 to 35 | 5,496 | 5,400 | ± 81 | 0.091 |  | 5,543^a^ | 5,616^a^ | 5,185^b^ | ± 98 | <0.001 |  | 0.595 |
| ^a,b^Means within a row with different superscripts differ significantly (*P* ≤ 0.05).  ^1^Values are least square means of 30 (limestone PS main effect) and 20 (Ca concentration main effect) replicate pens.  ^2^Limestone was ground using a 2-pair roller mill (Roskamp Champion Series 900-12, California Pellet Mill Co., Crawfordsville, IN) to achieve a PS of 910 µm (coarse) and 200 µm (fine).  ^3^Calcium concentration was a two-step, 0.10 percentage unit reduction from the primary breeder’s requirements for dietary Ca for each of the growth phases (Starter: Adequate (0.95 %), Reduced (0.85 %), Low (0.75 %); Grower: Adequate (0.85 %), Reduced (0.75 %), Low (0.65 %); Finisher: Adequate (0.75 %), Reduced (0.65 %), Low (0.55 %).  ^4^CLM = 95% confidence limit for the mean.  ^5^P = Phosphorus. | | | | | | | | | | | | |

| **Table S5**. Digestible essential and total amino acid intake (units/bird; DM basis) of YPM x Ross 708 male broilers provided diets varying in limestone particle size (PS) and calcium (Ca) concentration between d 17 to 21 and 27 to 35^1^. | | | | | | | | | | | | |
| --- | --- | --- | --- | --- | --- | --- | --- | --- | --- | --- | --- | --- |
|  | Main effect | | | | | | | | | |  | Interaction |
|  | Limestone PS^2^, µm (n = 30) | | | |  | Ca concentration^3^, (n = 20) | | | | |  | Limestone PS x  Ca concentration |
| Digestible nutrient intake | 910 | 200 | CLM^4^ | *P*-value |  | Adequate | Reduced | Low | CLM | *P*-value |  | *P*-value |
| Met, g |  |  |  |  |  |  |  |  |  |  |  |  |
| d 17 to 21 | 2.53 | 2.58 | ± 0.04 | 0.079 |  | 2.64 | 2.55 | 2.47 | ± 0.05 | <0.001 |  | <0.001 |
| d 27 to 35 | 10.28 | 10.88 | ± 0.16 | <0.001 |  | 10.25 | 11.11 | 10.38 | ± 0.20 | <0.001 |  | <0.001 |
| Met + Cys, g |  |  |  |  |  |  |  |  |  |  |  |  |
| d 17 to 21 | 3.48 | 3.58 | ± 0.07 | 0.042 |  | 3.64 | 3.51 | 3.44 | ± 0.09 | 0.005 |  | <0.001 |
| d 27 to 35 | 14.60 | 15.23 | ± 0.26 | 0.001 |  | 14.78 | 15.74 | 14.23 | ± 0.34 | <0.001 |  | 0.040 |
| Lys, g |  |  |  |  |  |  |  |  |  |  |  |  |
| d 17 to 21 | 4.81^b^ | 5.13^a^ | ± 0.10 | <0.001 |  | 5.16^a^ | 4.98^a^ | 4.77^b^ | ± 0.12 | <0.001 |  | 0.227 |
| d 27 to 35 | 20.11 | 20.47 | ± 0.47 | 0.276 |  | 19.73 | 21.37 | 19.79 | ± 0.57 | <0.001 |  | 0.030 |
| Thr, g |  |  |  |  |  |  |  |  |  |  |  |  |
| d 17 to 21 | 2.95 | 2.93 | ± 0.07 | 0.655 |  | 3.15 | 2.85 | 2.81 | ± 0.08 | <0.001 |  | <0.001 |
| d 27 to 35 | 11.68 | 12.13 | ± 0.29 | 0.023 |  | 11.47 | 12.26 | 11.98 | ± 0.35 | 0.007 |  | <0.001 |
| Val, g |  |  |  |  |  |  |  |  |  |  |  |  |
| d 17 to 21 | 3.66 | 3.73 | ± 0.08 | 0.220 |  | 3.84^a^ | 3.68^ab^ | 3.55^b^ | ± 0.10 | 0.001 |  | 0.274 |
| d 27 to 35 | 15.06 | 15.31 | ± 0.34 | 0.294 |  | 14.70 | 16.17 | 14.68 | ± 0.42 | <0.001 |  | 0.001 |
| Ile, g |  |  |  |  |  |  |  |  |  |  |  |  |
| d 17 to 21 | 3.21 | 3.32 | ± 0.07 | 0.035 |  | 3.42^a^ | 3.26^b^ | 3.12^b^ | ± 0.09 | <0.001 |  | 0.594 |
| d 27 to 35 | 13.54 | 13.81 | ± 0.30 | 0.200 |  | 13.21 | 14.65 | 13.16 | ± 0.36 | <0.001 |  | 0.002 |
| Arg, g |  |  |  |  |  |  |  |  |  |  |  |  |
| d 17 to 21 | 5.18 | 5.47 | ± 0.08 | <0.001 |  | 5.50^a^ | 5.39^a^ | 5.08^b^ | ± 0.10 | <0.001 |  | 0.130 |
| d 27 to 35 | 22.18 | 22.50 | ± 0.39 | 0.245 |  | 21.74 | 23.76 | 21.51 | ± 0.47 | <0.001 |  | 0.001 |
| Trp, g |  |  |  |  |  |  |  |  |  |  |  |  |
| d 17 to 21 | 0.99 | 1.04 | ± 0.02 | <0.001 |  | 1.07 | 1.02 | 0.95 | ± 0.02 | <0.001 |  | <0.001 |
| d 27 to 35 | 3.72 | 3.76 | ± 0.08 | 0.548 |  | 3.75^ab^ | 3.83^a^ | 3.65^b^ | ± 0.10 | 0.043 |  | 0.381 |
| Leu, g |  |  |  |  |  |  |  |  |  |  |  |  |
| d 17 to 21 | 6.36 | 6.54 | ± 0.13 | 0.053 |  | 6.60^a^ | 6.46^ab^ | 6.29^b^ | ± 0.17 | 0.035 |  | 0.750 |
| d 27 to 35 | 26.54 | 26.71 | ± 0.54 | 0.648 |  | 26.13^b^ | 28.34^a^ | 25.40^b^ | ± 0.65 | <0.001 |  | 0.079 |
| Phe, g |  |  |  |  |  |  |  |  |  |  |  |  |
| d 17 to 21 | 3.74^b^ | 3.89^a^ | ± 0.07 | 0.007 |  | 3.95^a^ | 3.80^ab^ | 3.69^b^ | ± 0.09 | 0.001 |  | 0.260 |
| d 27 to 35 | 15.74 | 15.94 | ± 0.33 | 0.393 |  | 15.53 | 16.86 | 15.14 | ± 0.41 | <0.001 |  | 0.016 |
| His, g |  |  |  |  |  |  |  |  |  |  |  |  |
| d 17 to 21 | 1.95^b^ | 2.04^a^ | ± 0.04 | 0.002 |  | 2.07^a^ | 2.00^a^ | 1.91^b^ | ± 0.05 | <0.001 |  | 0.273 |
| d 27 to 35 | 8.30 | 8.40 | ± 0.16 | 0.379 |  | 8.15 | 8.88 | 8.01 | ± 0.19 | <0.001 |  | 0.007 |
| Total AA, g |  |  |  |  |  |  |  |  |  |  |  |  |
| d 17 to 21 | 74.52^b^ | 77.32^a^ | ± 1.50 | 0.010 |  | 78.33^a^ | 75.82^ab^ | 73.61^b^ | ± 1.86 | 0.003 |  | 0.566 |
| d 27 to 35 | 312 | 316 | ± 6 | 0.379 |  | 307 | 334 | 301 | ± 8 | <0.001 |  | 0.012 |
| ^a,b^Means within a row with different superscripts differ significantly (*P* ≤ 0.05).  ^1^Values are least square means of 30 (limestone PS main effect) and 20 (Ca concentration main effect) replicate pens.  ^2^Limestone was ground using a 2-pair roller mill (Roskamp Champion Series 900-12, California Pellet Mill Co., Crawfordsville, IN) to achieve a PS of 910 µm (coarse) and 200 µm (fine).  ^3^Calcium concentration was a two-step, 0.10 percentage unit reduction from the primary breeder’s requirements for dietary Ca for each of the growth phases (Starter: Adequate (0.95 %), Reduced (0.85 %), Low (0.75 %); Grower: Adequate (0.85 %), Reduced (0.75 %), Low (0.65 %); Finisher: Adequate (0.75 %), Reduced (0.65 %), Low (0.55 %).  ^4^CLM = 95% confidence limit for the mean. | | | | | | | | | | | | |

| **Table S6**. Digestible non-essential amino acid intake (units/bird; DM basis) of YPM x Ross 708 male broilers provided diets varying in limestone particle size (PS) and calcium (Ca) concentration between d 17 to 21 and 27 to 35^1^. | | | | | | | | | | | | |
| --- | --- | --- | --- | --- | --- | --- | --- | --- | --- | --- | --- | --- |
|  | Main effect | | | | | | | | | |  | Interaction |
|  | Limestone PS^2^, µm (n = 30) | | | |  | Ca concentration^3^, (n = 20) | | | | |  | Limestone PS x  Ca concentration |
| Digestible nutrient intake | 910 | 200 | CLM^4^ | *P*-value |  | Adequate | Reduced | Low | CLM | *P*-value |  | *P*-value |
| Ala, g |  |  |  |  |  |  |  |  |  |  |  |  |
| d 17 to 21 | 3.60 | 3.69 | ± 0.08 | 0.149 |  | 3.71 | 3.66 | 3.57 | ± 0.10 | 0.124 |  | 0.843 |
| d 27 to 35 | 15.42 | 15.40 | ± 0.34 | 0.964 |  | 15.27^b^ | 16.25^a^ | 14.71^b^ | ± 0.42 | <0.001 |  | 0.246 |
| Asp, g |  |  |  |  |  |  |  |  |  |  |  |  |
| d 17 to 21 | 7.26^b^ | 7.64^a^ | ± 0.15 | <0.001 |  | 7.73^a^ | 7.42^b^ | 7.20^b^ | ± 0.19 | 0.001 |  | 0.169 |
| d 27 to 35 | 31.22 | 31.75 | ± 0.60 | 0.198 |  | 30.70 | 33.69 | 30.06 | ± 0.73 | <0.001 |  | <0.001 |
| Cys, g |  |  |  |  |  |  |  |  |  |  |  |  |
| d 17 to 21 | 0.95 | 1.00 | ± 0.03 | 0.002 |  | 1.00 | 0.99 | 0.93 | ± 0.03 | 0.005 |  | <0.001 |
| d 27 to 35 | 4.25 | 4.35 | ± 0.13 | 0.266 |  | 4.35^b^ | 4.65^a^ | 3.89^c^ | ± 0.16 | <0.001 |  | 0.146 |
| Glu, g |  |  |  |  |  |  |  |  |  |  |  |  |
| d 17 to 21 | 14.29^b^ | 14.97^a^ | ± 0.22 | <0.001 |  | 15.04^a^ | 14.70^a^ | 14.15^b^ | ± 0.27 | <0.001 |  | 0.612 |
| d 27 to 35 | 60.17 | 60.98 | ± 0.96 | 0.229 |  | 59.71 | 64.48 | 57.53 | ± 1.20 | <0.001 |  | 0.011 |
| Gly, g |  |  |  |  |  |  |  |  |  |  |  |  |
| d 17 to 21 | 2.79^b^ | 2.91^a^ | ± 0.06 | 0.011 |  | 2.95^a^ | 2.86^a^ | 2.72^b^ | ± 0.08 | 0.001 |  | 0.343 |
| d 27 to 35 | 12.00 | 12.06 | ± 0.29 | 0.742 |  | 11.69 | 12.79 | 11.61 | ± 0.35 | <0.001 |  | 0.028 |
| Pro, g |  |  |  |  |  |  |  |  |  |  |  |  |
| d 17 to 21 | 4.21^b^ | 4.33^a^ | ± 0.08 | 0.047 |  | 4.39^a^ | 4.29^ab^ | 4.14^b^ | ± 0.10 | 0.003 |  | 0.714 |
| d 27 to 35 | 17.31 | 17.70 | ± 0.31 | 0.083 |  | 17.51^b^ | 18.58^a^ | 16.41^c^ | ± 0.40 | <0.001 |  | 0.106 |
| Ser, g |  |  |  |  |  |  |  |  |  |  |  |  |
| d 17 to 21 | 3.03^b^ | 3.29^a^ | ± 0.06 | <0.001 |  | 3.21 | 3.16 | 3.12 | ± 0.08 | 0.319 |  | 0.454 |
| d 27 to 35 | 13.38 | 13.21 | ± 0.26 | 0.348 |  | 13.44 | 14.13 | 12.32 | ± 0.32 | <0.001 |  | 0.001 |
| Tyr, g |  |  |  |  |  |  |  |  |  |  |  |  |
| d 17 to 21 | 2.46 | 2.55 | ± 0.05 | 0.018 |  | 2.53 | 2.55 | 2.44 | ± 0.06 | 0.032 |  | 0.010 |
| d 27 to 35 | 10.27 | 10.28 | ± 0.22 | 0.970 |  | 10.21 | 11.03 | 9.58 | ± 0.28 | <0.001 |  | 0.019 |
| ^a-c^Means within a row with different superscripts differ significantly (*P* ≤ 0.05).  ^1^Values are least square means of 30 (limestone PS main effect) and 20 (Ca concentration main effect) replicate pens.  ^2^Limestone was ground using a 2-pair roller mill (Roskamp Champion Series 900-12, California Pellet Mill Co., Crawfordsville, IN) to achieve a PS of 910 µm (coarse) and 200 µm (fine).  ^3^Calcium concentration was a two-step, 0.10 percentage unit reduction from the primary breeder’s requirements for dietary Ca for each of the growth phases (Starter: Adequate (0.95 %), Reduced (0.85 %), Low (0.75 %); Grower: Adequate (0.85 %), Reduced (0.75 %), Low (0.65 %); Finisher: Adequate (0.75 %), Reduced (0.65 %), Low (0.55 %).  ^4^CLM = 95% confidence limit for the mean. | | | | | | | | | | | | |

| **Table S7**. Performance and mortality responses to calcium (Ca) concentrations as a main effect and within each limestone particle size (PS) group as assessed by orthogonal polynomial contrasts (*P*-values)^1^. | | | | | | | | |
| --- | --- | --- | --- | --- | --- | --- | --- | --- |
|  | Ca concentration | |  | Limestone particle size^2^, µm | | | | |
|  |  |  |  | 910 | |  | 200 | |
| Measurements | Linear | Quadratic |  | Linear | Quadratic |  | Linear | Quadratic |
| BW, g/bird |  |  |  |  |  |  |  |  |
| d 17 | <0.001 | 0.001 |  | <0.001 | 0.001 |  | <0.001 | 0.172 |
| d 21 | <0.001 | 0.003 |  | <0.001 | 0.002 |  | <0.001 | 0.279 |
| d 26 | <0.001 | 0.030 |  | <0.001 | 0.120 |  | <0.001 | 0.121 |
| d 35 | <0.001 | <0.001 |  | <0.001 | 0.004 |  | <0.001 | 0.001 |
| Feed intake, g/bird |  |  |  |  |  |  |  |  |
| d 1 to 17 | 0.003 | 0.047 |  | 0.019 | 0.129 |  | 0.045 | 0.187 |
| d 17 to 21 | 0.004 | 0.066 |  | 0.041 | 0.304 |  | 0.029 | 0.109 |
| d 1 to 21 | <0.001 | 0.024 |  | <0.001 | 0.024 |  | 0.003 | 0.342 |
| d 18 to 26 | <0.001 | 0.010 |  | 0.006 | 0.080 |  | 0.002 | 0.049 |
| d 1 to 26 | <0.001 | 0.005 |  | 0.003 | 0.045 |  | 0.020 | 0.040 |
| d 27 to 35 | <0.001 | <0.001 |  | 0.119 | 0.009 |  | <0.001 | 0.004 |
| d 1 to 35 | <0.001 | <0.001 |  | <0.001 | <0.001 |  | <0.001 | 0.003 |
| FCR, g:g |  |  |  |  |  |  |  |  |
| d 1 to 17 | <0.001 | 0.017 |  | <0.001 | 0.002 |  | <0.001 | 0.755 |
| d 1 to 21 | <0.001 | 0.138 |  | <0.001 | 0.106 |  | <0.001 | 0.632 |
| d 18 to 26 | 0.190 | 0.610 |  | 0.122 | 0.659 |  | 0.772 | 0.780 |
| d 1 to 26 | <0.001 | 0.376 |  | <0.001 | 0.426 |  | <0.001 | 0.651 |
| d 27 to 35 | <0.001 | 0.001 |  | <0.001 | 0.328 |  | 0.004 | <0.001 |
| d 1 to 35 | <0.001 | 0.002 |  | <0.001 | 0.080 |  | 0.001 | 0.007 |
| Mortality, % |  |  |  |  |  |  |  |  |
| d 1 to 17 | 0.911 | 0.948 |  | 0.823 | 0.899 |  | 0.705 | 0.827 |
| d 1 to 21 | 0.977 | 0.374 |  | 0.712 | 0.569 |  | 0.743 | 0.490 |
| d 18 to 26 | 0.828 | 0.428 |  | 0.682 | 0.068 |  | 0.474 | 0.466 |
| d 1 to 26 | 0.944 | 0.462 |  | 0.898 | 0.219 |  | 0.820 | 0.845 |
| d 27 to 35 | 0.595 | 0.016 |  | 0.247 | 0.008 |  | 0.682 | 0.431 |
| d 1 to 35 | 0.891 | 0.163 |  | 0.535 | 0.306 |  | 0.669 | 0.338 |
| ^1^Statistical significance was considered at *P* ≤ 0.05; Orthogonal polynomial contrasts were performed for the equally spaced Ca concentrations for the main effect and within each limestone PS group.  ^2^Limestone was ground using a 2-pair roller mill (Roskamp Champion Series 900-12, California Pellet Mill Co., Crawfordsville, IN) to achieve a PS of 910 µm (coarse) and 200 µm (fine). | | | | | | | | |

| **Table S8**. Tibia mineralization responses to calcium (Ca) concentrations as a main effect and within each limestone particle size (PS) group as assessed by orthogonal polynomial contrasts (*P*-values)^1^. | | | | | | | | |
| --- | --- | --- | --- | --- | --- | --- | --- | --- |
|  | Ca concentration | |  | Limestone particle size^2^, µm | | | | |
|  |  |  |  | 910 | |  | 200 | |
| Measurements | Linear | Quadratic |  | Linear | Quadratic |  | Linear | Quadratic |
| Tibia weight, g |  |  |  |  |  |  |  |  |
| d 21 | <0.001 | 0.145 |  | <0.001 | 0.095 |  | 0.001 | 0.727 |
| d 35 | <0.001 | 0.019 |  | <0.001 | 0.400 |  | 0.009 | 0.007 |
| Shear strength, N^3^ |  |  |  |  |  |  |  |  |
| d 21 | <0.001 | 0.927 |  | <0.001 | 0.407 |  | <0.001 | 0.484 |
| d 35 | <0.001 | 0.003 |  | <0.001 | 0.038 |  | <0.001 | 0.028 |
| Tibia ash, % |  |  |  |  |  |  |  |  |
| d 21 | <0.001 | 0.923 |  | 0.001 | 0.413 |  | <0.001 | 0.525 |
| d 35 | <0.001 | 0.077 |  | 0.017 | 0.290 |  | <0.001 | 0.143 |
| ^1^Statistical significance was considered at *P* ≤ 0.05; Orthogonal polynomial contrasts were performed for the equally spaced Ca concentrations for the main effect and within each limestone PS group.  ^2^Limestone was ground using a 2-pair roller mill (Roskamp Champion Series 900-12, California Pellet Mill Co., Crawfordsville, IN) to achieve a PS of 910 µm (coarse) and 200 µm (fine).  ^3^N = Newtons. | | | | | | | | |

| **Table S9**. Nutrient digestibility and apparent ileal digestible energy (AIDE) responses to calcium (Ca) concentrations as a main effect and within each limestone particle size (PS) group as assessed by orthogonal polynomial contrasts (*P*-values)^1^. | | | | | | | | |
| --- | --- | --- | --- | --- | --- | --- | --- | --- |
|  | Ca concentration | |  | Limestone particle size^2^, µm | | | | |
|  |  |  |  | 910 | |  | 200 | |
| Measurements | Linear | Quadratic |  | Linear | Quadratic |  | Linear | Quadratic |
| CP digestibility, % |  |  |  |  |  |  |  |  |
| d 21 | 0.608 | 0.877 |  | 0.082 | 0.396 |  | 0.341 | 0.538 |
| d 35 | 0.964 | 0.259 |  | 0.391 | 0.253 |  | 0.473 | 0.640 |
| Fat digestibility, % |  |  |  |  |  |  |  |  |
| d 21 | 0.127 | 0.986 |  | 0.012 | 0.929 |  | 0.680 | 0.937 |
| d 35 | 0.830 | 0.458 |  | 0.975 | 0.317 |  | 0.785 | 0.992 |
| Ca digestibility, % |  |  |  |  |  |  |  |  |
| d 21 | <0.001 | 0.033 |  | <0.001 | 0.002 |  | <0.001 | 0.811 |
| d 35 | 0.001 | 0.292 |  | 0.054 | 0.109 |  | 0.002 | 0.886 |
| P^3^ digestibility, % |  |  |  |  |  |  |  |  |
| d 21 | <0.001 | 0.449 |  | <0.001 | 0.350 |  | <0.001 | 0.041 |
| d 35 | <0.001 | 0.976 |  | 0.001 | 0.472 |  | <0.001 | 0.511 |
| AIDE^4^, kcal/kg |  |  |  |  |  |  |  |  |
| d 21 | 0.128 | 0.057 |  | 0.008 | 0.008 |  | 0.525 | 0.981 |
| d 35 | <0.001 | 0.182 |  | 0.001 | 0.820 |  | 0.034 | 0.109 |
| ^1^Statistical significance was considered at *P* ≤ 0.05; Orthogonal polynomial contrasts were performed for the equally spaced Ca concentrations for the main effect and within each limestone PS group.  ^2^Limestone was ground using a 2-pair roller mill (Roskamp Champion Series 900-12, California Pellet Mill Co., Crawfordsville, IN) to achieve a PS of 910 µm (coarse) and 200 µm (fine).  ^3^P = Phosphorus.  ^4^On a DM basis. | | | | | | | | |

| **Table S10**. Apparent essential and total ileal amino acid (AA) digestibility responses to calcium (Ca) concentrations as a main effect and within each limestone particle size (PS) group as assessed by orthogonal polynomial contrasts (*P*-values)^1^. | | | | | | | | | |
| --- | --- | --- | --- | --- | --- | --- | --- | --- | --- |
|  | Ca concentration | |  | Limestone particle size^2^, µm | | | | |  |
|  |  |  |  | 910 | |  | 200 | |  |
| Digestibility, % | Linear | Quadratic |  | Linear | Quadratic |  | Linear | Quadratic |  |
| Met |  |  |  |  |  |  |  |  |  |
| d 21 | 0.077 | 0.588 |  | 0.004 | 0.246 |  | 0.662 | 0.057 |  |
| d 35 | 0.307 | 0.541 |  | 0.477 | 0.960 |  | 0.460 | 0.423 |  |
| Met + Cys |  |  |  |  |  |  |  |  |  |
| d 21 | 0.976 | 0.861 |  | 0.009 | 0.065 |  | 0.010 | 0.107 |  |
| d 35 | 0.141 | 0.109 |  | 0.192 | 0.391 |  | 0.417 | 0.158 |  |
| Lys |  |  |  |  |  |  |  |  |  |
| d 21 | 0.265 | 0.589 |  | 0.410 | 0.483 |  | 0.449 | 0.146 |  |
| d 35 | 0.466 | 0.511 |  | 0.790 | 0.626 |  | 0.222 | 0.658 |  |
| Thr |  |  |  |  |  |  |  |  |  |
| d 21 | 0.972 | 0.230 |  | 0.323 | 0.926 |  | 0.347 | 0.080 |  |
| d 35 | 0.492 | 0.688 |  | 0.285 | 0.215 |  | 0.070 | 0.529 |  |
| Val |  |  |  |  |  |  |  |  |  |
| d 21 | 0.337 | 0.772 |  | 0.299 | 0.449 |  | 0.751 | 0.245 |  |
| d 35 | 0.702 | 0.251 |  | 0.386 | 0.527 |  | 0.195 | 0.323 |  |
| Ile |  |  |  |  |  |  |  |  |  |
| d 21 | 0.499 | 0.770 |  | 0.305 | 0.465 |  | 0.941 | 0.254 |  |
| d 35 | 0.735 | 0.169 |  | 0.377 | 0.336 |  | 0.210 | 0.322 |  |
| Arg |  |  |  |  |  |  |  |  |  |
| d 21 | 0.380 | 0.933 |  | 0.456 | 0.603 |  | 0.619 | 0.523 |  |
| d 35 | 0.450 | 0.209 |  | 0.628 | 0.337 |  | 0.148 | 0.409 |  |
| Trp |  |  |  |  |  |  |  |  |  |
| d 21 | 0.396 | 0.795 |  | 0.815 | 0.842 |  | 0.338 | 0.573 |  |
| d 35 | 0.550 | 0.794 |  | 0.727 | 0.914 |  | 0.263 | 0.640 |  |
| Leu |  |  |  |  |  |  |  |  |  |
| d 21 | 0.173 | 0.850 |  | 0.103 | 0.458 |  | 0.770 | 0.314 |  |
| d 35 | 0.972 | 0.221 |  | 0.408 | 0.303 |  | 0.481 | 0.477 |  |
| Phe |  |  |  |  |  |  |  |  |  |
| d 21 | 0.233 | 0.631 |  | 0.234 | 0.532 |  | 0.616 | 0.195 |  |
| d 35 | 0.919 | 0.165 |  | 0.347 | 0.225 |  | 0.322 | 0.444 |  |
| His |  |  |  |  |  |  |  |  |  |
| d 21 | 0.727 | 0.852 |  | 0.598 | 0.335 |  | 0.973 | 0.482 |  |
| d 35 | 0.702 | 0.103 |  | 0.154 | 0.201 |  | 0.432 | 0.295 |  |
| Total AA |  |  |  |  |  |  |  |  |  |
| d 21 | 0.327 | 0.844 |  | 0.246 | 0.431 |  | 0.822 | 0.288 |  |
| d 35 | 0.978 | 0.163 |  | 0.302 | 0.225 |  | 0.367 | 0.438 |  |
| ^1^Statistical significance was considered at *P* ≤ 0.05; Orthogonal polynomial contrasts were performed for the equally spaced Ca concentrations for the main effect and within each limestone PS group.  ^2^Limestone was ground using a 2-pair roller mill (Roskamp Champion Series 900-12, California Pellet Mill Co., Crawfordsville, IN) to achieve a PS of 910 µm (coarse) and 200 µm (fine). | | | | | | | | | |

| **Table S11**. Apparent non-essential amino acid digestibility responses to calcium (Ca) concentrations as a main effect and within each limestone particle size (PS) group as assessed by orthogonal polynomial contrasts (*P*-values)^1^. | | | | | | | | |
| --- | --- | --- | --- | --- | --- | --- | --- | --- |
|  | Ca concentration | |  | Limestone particle size^2^, µm | | | | |
|  |  |  |  | 910 | |  | 200 | |
| Digestibility, % | Linear | Quadratic |  | Linear | Quadratic |  | Linear | Quadratic |
| Ala |  |  |  |  |  |  |  |  |
| d 21 | 0.094 | 0.891 |  | 0.070 | 0.407 |  | 0.573 | 0.307 |
| d 35 | 0.970 | 0.358 |  | 0.418 | 0.504 |  | 0.494 | 0.526 |
| Asp |  |  |  |  |  |  |  |  |
| d 21 | 0.592 | 0.921 |  | 0.566 | 0.428 |  | 0.854 | 0.351 |
| d 35 | 0.961 | 0.070 |  | 0.183 | 0.089 |  | 0.202 | 0.375 |
| Cys |  |  |  |  |  |  |  |  |
| d 21 | 0.272 | 0.299 |  | 0.082 | 0.018 |  | 0.003 | 0.375 |
| d 35 | 0.001 | 0.025 |  | 0.002 | 0.062 |  | 0.054 | 0.180 |
| Glu |  |  |  |  |  |  |  |  |
| d 21 | 0.312 | 0.420 |  | 0.178 | 0.920 |  | 0.939 | 0.208 |
| d 35 | 0.864 | 0.128 |  | 0.250 | 0.173 |  | 0.414 | 0.418 |
| Gly |  |  |  |  |  |  |  |  |
| d 21 | 0.575 | 0.807 |  | 0.380 | 0.188 |  | 0.931 | 0.329 |
| d 35 | 0.886 | 0.177 |  | 0.238 | 0.280 |  | 0.378 | 0.400 |
| Pro |  |  |  |  |  |  |  |  |
| d 21 | 0.668 | 0.768 |  | 0.260 | 0.282 |  | 0.599 | 0.508 |
| d 35 | 0.441 | 0.042 |  | 0.454 | 0.041 |  | 0.725 | 0.390 |
| Ser |  |  |  |  |  |  |  |  |
| d 21 | 0.257 | 0.615 |  | 0.096 | 0.127 |  | 0.994 | 0.406 |
| d 35 | 0.077 | 0.134 |  | 0.048 | 0.073 |  | 0.527 | 0.721 |
| Tyr |  |  |  |  |  |  |  |  |
| d 21 | 0.030 | 0.688 |  | 0.130 | 0.729 |  | 0.113 | 0.825 |
| d 35 | 0.390 | 0.358 |  | 0.303 | 0.185 |  | 0.846 | 0.979 |
| ^1^Statistical significance was considered at *P* ≤ 0.05; Orthogonal polynomial contrasts were performed for the equally spaced Ca concentrations for the main effect and within each limestone PS group.  ^2^Limestone was ground using a 2-pair roller mill (Roskamp Champion Series 900-12, California Pellet Mill Co., Crawfordsville, IN) to achieve a PS of 910 µm (coarse) and 200 µm (fine). | | | | | | | | |

| **Table S12**. Digestible nutrient intake (units/bird; DM basis) responses to calcium (Ca) concentrations as a main effect and within each limestone particle size (PS) group as assessed by orthogonal polynomial contrasts (*P*-values)^1^. | | | | | | | | |
| --- | --- | --- | --- | --- | --- | --- | --- | --- |
|  | Ca concentration | |  | Limestone particle size^2^, µm | | | | |
|  |  |  |  | 910 | |  | 200 | |
| Digestible nutrient intake | Linear | Quadratic |  | Linear | Quadratic |  | Linear | Quadratic |
| CP, g |  |  |  |  |  |  |  |  |
| d 17 to 21 | <0.001 | 0.129 |  | 0.086 | 0.537 |  | <0.001 | 0.129 |
| d 27 to 35 | 0.068 | <0.001 |  | 0.024 | <0.001 |  | 0.739 | 0.036 |
| Fat, g |  |  |  |  |  |  |  |  |
| d 17 to 21 | 0.916 | 0.083 |  | 0.618 | 0.088 |  | 0.727 | 0.473 |
| d 27 to 35 | 0.008 | 0.142 |  | 0.001 | 0.175 |  | 0.658 | 0.001 |
| Ca, g |  |  |  |  |  |  |  |  |
| d 17 to 21 | 0.421 | 0.018 |  | 0.003 | <0.001 |  | <0.001 | 0.082 |
| d 27 to 35 | <0.001 | 0.012 |  | <0.001 | <0.001 |  | <0.001 | 0.769 |
| P^3^, g |  |  |  |  |  |  |  |  |
| d 17 to 21 | 0.502 | 0.602 |  | 0.072 | 0.388 |  | 0.346 | 0.905 |
| d 27 to 35 | 0.001 | 0.695 |  | <0.001 | 0.721 |  | 0.373 | 0.363 |
| Calorie, kcal |  |  |  |  |  |  |  |  |
| d 17 to 21 | 0.369 | 0.004 |  | 0.514 | 0.008 |  | 0.058 | 0.141 |
| d 27 to 35 | <0.001 | <0.001 |  | 0.001 | 0.021 |  | 0.001 | 0.001 |
| ^1^Statistical significance was considered at *P* ≤ 0.05; Orthogonal polynomial contrasts were performed for the equally spaced Ca concentrations for the main effect and within each limestone PS group.  ^2^Limestone was ground using a 2-pair roller mill (Roskamp Champion Series 900-12, California Pellet Mill Co., Crawfordsville, IN) to achieve a PS of 910 µm (coarse) and 200 µm (fine).  ^3^P = Phosphorus. | | | | | | | | |

| **Table S13**. Digestible essential and total amino acid (AA) intake (units/bird; DM basis) responses to calcium (Ca) concentrations as a main effect and within each limestone particle size (PS) group as assessed by orthogonal polynomial contrasts (*P*-values)^1^. | | | | | | | | | |
| --- | --- | --- | --- | --- | --- | --- | --- | --- | --- |
|  | Ca concentration | |  | Limestone particle size^2^, µm | | | | |  |
|  |  |  |  | 910 | |  | 200 | |  |
| Digestible nutrient intake | Linear | Quadratic |  | Linear | Quadratic |  | Linear | Quadratic |  |
| Met, g |  |  |  |  |  |  |  |  |  |
| d 17 to 21 | <0.001 | 0.800 |  | 0.036 | 0.012 |  | <0.001 | 0.006 |  |
| d 27 to 35 | 0.372 | <0.001 |  | 0.001 | 0.150 |  | 0.063 | <0.001 |  |
| Met + Cys, g |  |  |  |  |  |  |  |  |  |
| d 17 to 21 | 0.001 | 0.584 |  | 0.045 | 0.024 |  | <0.001 | 0.004 |  |
| d 27 to 35 | 0.020 | <0.001 |  | 0.248 | 0.009 |  | 0.030 | <0.001 |  |
| Lys, g |  |  |  |  |  |  |  |  |  |
| d 17 to 21 | <0.001 | 0.770 |  | <0.001 | 0.493 |  | 0.026 | 0.795 |  |
| d 27 to 35 | 0.881 | <0.001 |  | 0.118 | <0.001 |  | 0.093 | 0.026 |  |
| Thr, g |  |  |  |  |  |  |  |  |  |
| d 17 to 21 | <0.001 | 0.011 |  | <0.001 | 0.102 |  | 0.658 | 0.043 |  |
| d 27 to 35 | 0.041 | 0.013 |  | 0.011 | <0.001 |  | <0.001 | 0.165 |  |
| Val, g |  |  |  |  |  |  |  |  |  |
| d 17 to 21 | <0.001 | 0.800 |  | <0.001 | 0.589 |  | 0.056 | 0.370 |  |
| d 27 to 35 | 0.949 | <0.001 |  | 0.006 | <0.001 |  | 0.012 | <0.001 |  |
| Ile, g |  |  |  |  |  |  |  |  |  |
| d 17 to 21 | <0.001 | 0.804 |  | <0.001 | 0.668 |  | 0.003 | 0.433 |  |
| d 27 to 35 | 0.864 | <0.001 |  | 0.006 | <0.001 |  | 0.016 | <0.001 |  |
| Arg, g |  |  |  |  |  |  |  |  |  |
| d 17 to 21 | <0.001 | 0.119 |  | <0.001 | 0.308 |  | 0.009 | 0.231 |  |
| d 27 to 35 | 0.484 | <0.001 |  | 0.007 | <0.001 |  | 0.097 | 0.001 |  |
| Trp, g |  |  |  |  |  |  |  |  |  |
| d 17 to 21 | <0.001 | 0.707 |  | <0.001 | 0.002 |  | <0.001 | <0.001 |  |
| d 27 to 35 | 0.141 | 0.036 |  | 0.093 | 0.430 |  | 0.687 | 0.029 |  |
| Leu, g |  |  |  |  |  |  |  |  |  |
| d 17 to 21 | 0.010 | 0.819 |  | 0.034 | 0.536 |  | 0.116 | 0.766 |  |
| d 27 to 35 | 0.120 | <0.001 |  | 0.007 | <0.001 |  | 0.621 | <0.001 |  |
| Phe, g |  |  |  |  |  |  |  |  |  |
| d 17 to 21 | <0.001 | 0.768 |  | <0.001 | 0.631 |  | 0.069 | 0.370 |  |
| d 27 to 35 | 0.177 | <0.001 |  | 0.008 | <0.001 |  | 0.444 | 0.002 |  |
| His, g |  |  |  |  |  |  |  |  |  |
| d 17 to 21 | <0.001 | 0.592 |  | <0.001 | 0.281 |  | 0.020 | 0.740 |  |
| d 27 to 35 | 0.284 | <0.001 |  | 0.002 | <0.001 |  | 0.148 | <0.001 |  |
| Total AA, g |  |  |  |  |  |  |  |  |  |
| d 17 to 21 | 0.001 | 0.891 |  | 0.003 | 0.646 |  | 0.047 | 0.517 |  |
| d 27 to 35 | 0.290 | <0.001 |  | 0.007 | <0.001 |  | 0.230 | 0.001 |  |
| ^1^Statistical significance was considered at *P* ≤ 0.05; Orthogonal polynomial contrasts were performed for the equally spaced Ca concentrations for the main effect and within each limestone PS group.  ^2^Limestone was ground using a 2-pair roller mill (Roskamp Champion Series 900-12, California Pellet Mill Co., Crawfordsville, IN) to achieve a PS of 910 µm (coarse) and 200 µm (fine). | | | | | | | | | |

| **Table S14**. Digestible non-essential amino acid intake (units/bird; DM basis) responses to calcium (Ca) concentrations as a main effect and within each limestone particle size (PS) group as assessed by orthogonal polynomial contrasts (*P*-values)^1^. | | | | | | | | |
| --- | --- | --- | --- | --- | --- | --- | --- | --- |
|  | Ca concentration | |  | Limestone particle size^2^, µm | | | | |
|  |  |  |  | 910 | |  | 200 | |
| Digestible nutrient intake | Linear | Quadratic |  | Linear | Quadratic |  | Linear | Quadratic |
| Ala, g |  |  |  |  |  |  |  |  |
| d 17 to 21 | 0.045 | 0.719 |  | 0.190 | 0.518 |  | 0.121 | 0.891 |
| d 27 to 35 | 0.064 | <0.001 |  | 0.013 | 0.001 |  | 0.894 | 0.001 |
| Asp, g |  |  |  |  |  |  |  |  |
| d 17 to 21 | <0.001 | 0.690 |  | <0.001 | 0.597 |  | 0.087 | 0.284 |
| d 27 to 35 | 0.210 | <0.001 |  | 0.001 | <0.001 |  | 0.101 | 0.002 |
| Cys, g |  |  |  |  |  |  |  |  |
| d 17 to 21 | 0.002 | 0.291 |  | 0.214 | 0.011 |  | <0.001 | 0.269 |
| d 27 to 35 | <0.001 | <0.001 |  | <0.001 | <0.001 |  | 0.034 | 0.007 |
| Glu, g |  |  |  |  |  |  |  |  |
| d 17 to 21 | <0.001 | 0.534 |  | <0.001 | 0.647 |  | 0.013 | 0.671 |
| d 27 to 35 | 0.011 | <0.001 |  | <0.001 | <0.001 |  | 0.928 | <0.001 |
| Gly, g |  |  |  |  |  |  |  |  |
| d 17 to 21 | <0.001 | 0.557 |  | 0.003 | 0.166 |  | 0.014 | 0.565 |
| d 27 to 35 | 0.758 | <0.001 |  | 0.041 | <0.001 |  | 0.124 | 0.003 |
| Pro, g |  |  |  |  |  |  |  |  |
| d 17 to 21 | 0.001 | 0.662 |  | 0.009 | 0.384 |  | 0.024 | 0.802 |
| d 27 to 35 | <0.001 | <0.001 |  | <0.001 | <0.001 |  | 0.203 | <0.001 |
| Ser, g |  |  |  |  |  |  |  |  |
| d 17 to 21 | 0.134 | 0.933 |  | 0.197 | 0.410 |  | 0.393 | 0.356 |
| d 27 to 35 | <0.001 | <0.001 |  | <0.001 | <0.001 |  | 0.002 | 0.093 |
| Tyr, g |  |  |  |  |  |  |  |  |
| d 17 to 21 | 0.041 | 0.080 |  | 0.001 | 0.794 |  | 0.622 | 0.028 |
| d 27 to 35 | 0.002 | <0.001 |  | 0.001 | <0.001 |  | 0.304 | 0.003 |
| ^1^Statistical significance was considered at *P* ≤ 0.05; Orthogonal polynomial contrasts were performed for the equally spaced Ca concentrations for the main effect and within each limestone PS group.  ^2^Limestone was ground using a 2-pair roller mill (Roskamp Champion Series 900-12, California Pellet Mill Co., Crawfordsville, IN) to achieve a PS of 910 µm (coarse) and 200 µm (fine). | | | | | | | | |


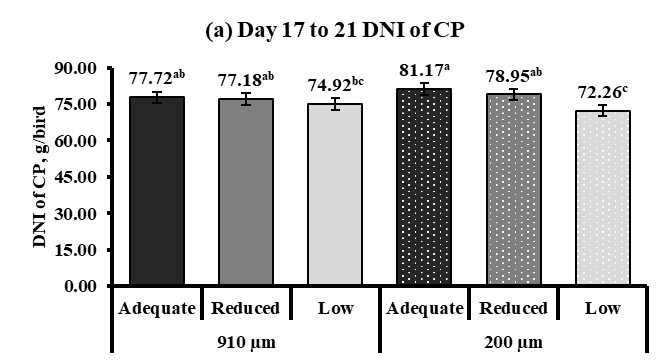

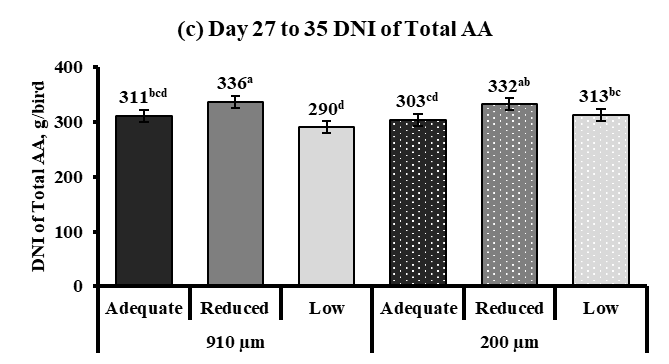

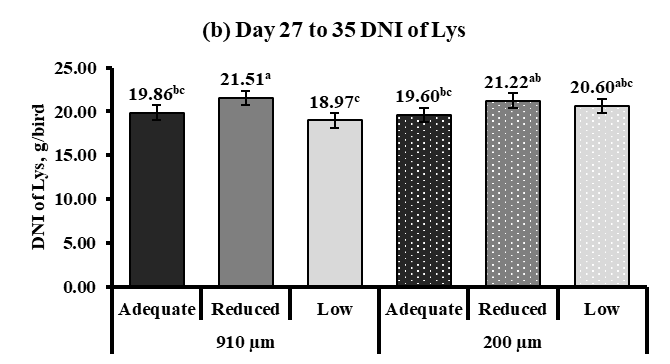


| **Figure S1**. Interaction effects^1^ of varying limestone particle size (910 and 200 µm) and Ca concentrations (adequate, reduced, and low) fed to YPM x Ross 708 male broilers on (**a**) d 17 to 21 DNI of CP (*P* = 0.031; CLM ± 2.40), (**b**) d 27 to 35 DNI of Lys (*P* = 0.030; CLM ± 0.83), and (**c**) d 27 to 35 DNI of total AA (*P* = 0.012; CLM ± 11). ^a-d^Means with different superscripts differ significantly (*P* ≤ 0.05). ^1^Interaction values are least square means of 10 replicate pens. |
| --- |


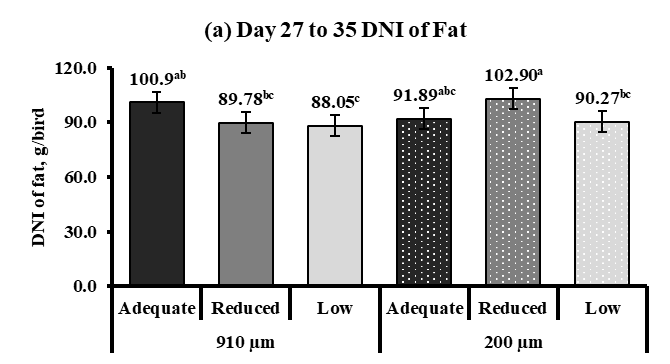

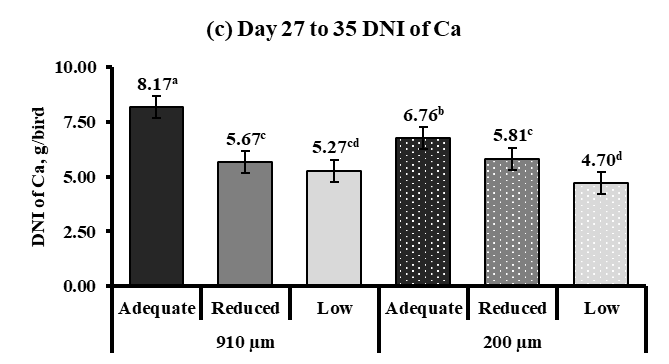

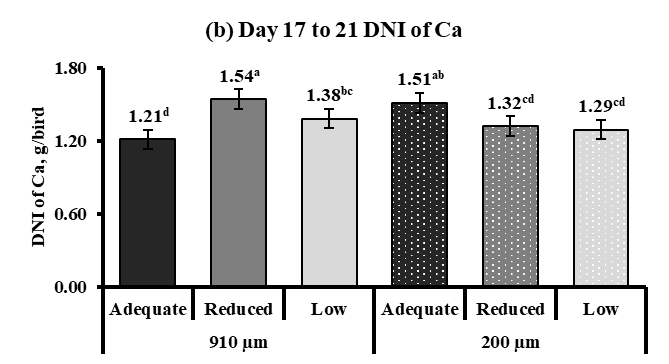


| **Figure S2**. Interaction effects^1^ of varying limestone particle size (910 and 200 µm) and Ca concentrations (adequate, reduced, and low) fed to YPM x Ross 708 male broilers on (**a**) d 27 to 35 DNI of fat (*P* = 0.001; CLM ± 5.77), (**b**) d 17 to 21 DNI of Ca (*P* < 0.001; CLM ± 0.08), and (**c**) d 27 to 35 DNI of Ca (*P* = 0.005; CLM ± 0.51). ^a-d^Means with different superscripts differ significantly (*P* ≤ 0.05). ^1^Interaction values are least square means of 10 replicate pens. |
| --- |


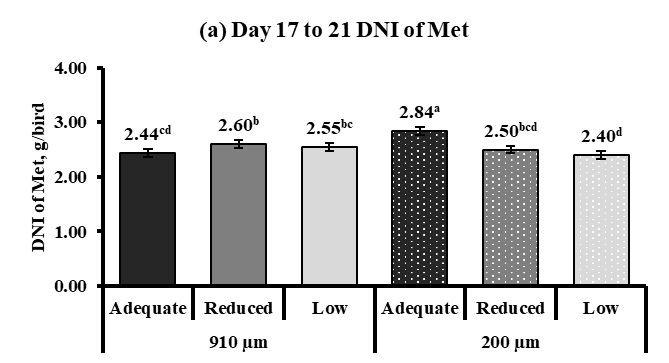

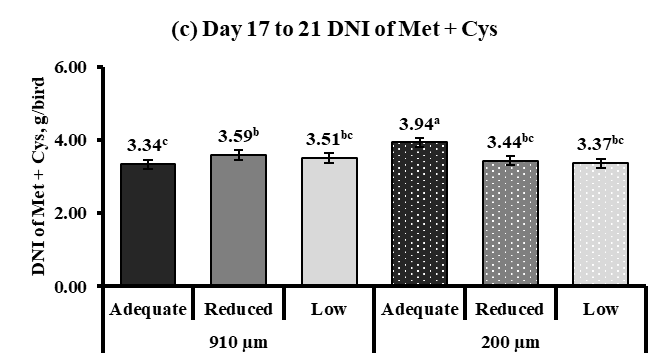

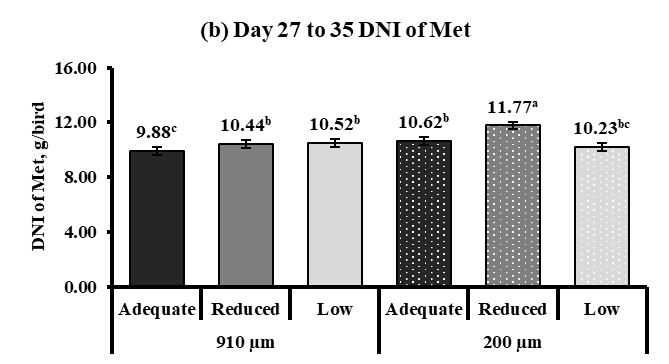

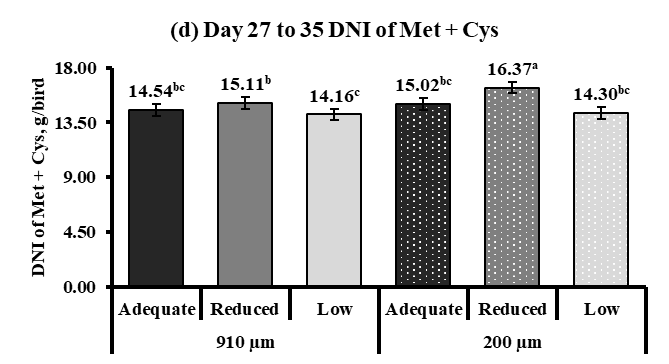


| **Figure S3**. Interaction effects^1^ of varying limestone particle size (910 and 200 µm) and Ca concentrations (adequate, reduced, and low) fed to YPM x Ross 708 male broilers on (**a**) d 17 to 21 DNI of Met (*P* < 0.001; CLM ± 0.07), (**b**) d 27 to 35 DNI of Met (*P* < 0.001; CLM ± 0.29), (**c**) d 17 to 21 DNI of Met + Cys (*P* < 0.001; CLM ± 0.13), and (**d**) d 27 to 35 DNI of Met + Cys (*P* = 0.040; CLM ± 0.48). ^a-d^Means with different superscripts differ significantly (*P* ≤ 0.05). ^1^Interaction values are least square means of 10 replicate pens. |
| --- |


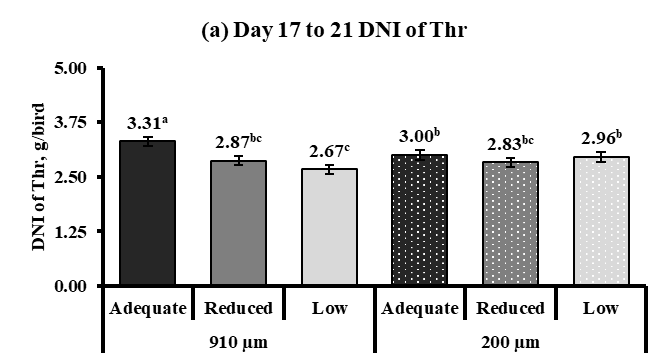

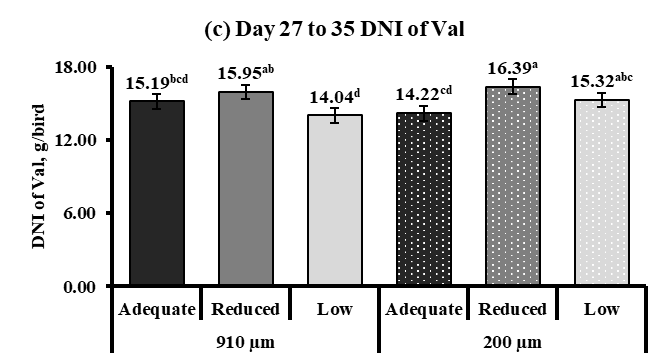

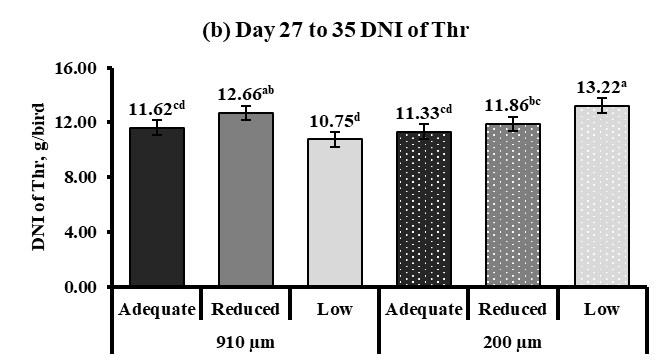

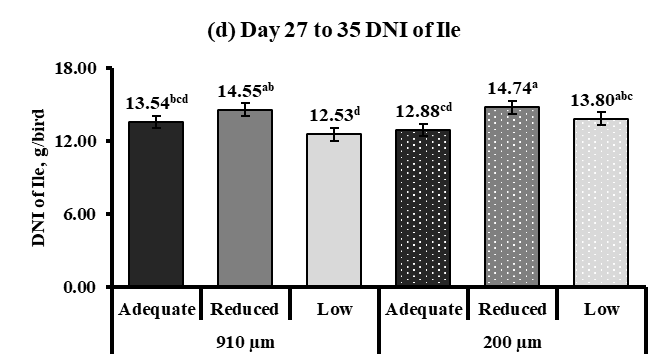


| **Figure S4**. Interaction effects^1^ of varying limestone particle size (910 and 200 µm) and Ca concentrations (adequate, reduced, and low) fed to YPM x Ross 708 male broilers on (**a**) d 17 to 21 DNI of Thr (*P* < 0.001; CLM ± 0.11), (**b**) d 27 to 35 DNI of Thr (*P* < 0.001; CLM ± 0.52), (**c**) d 27 to 35 DNI of Val (*P* = 0.001; CLM ± 0.60), and (**d**) d 27 to 35 DNI of Ile (*P* = 0.002; CLM ± 0.52). ^a-d^Means with different superscripts differ significantly (*P* ≤ 0.05). ^1^Interaction values are least square means of 10 replicate pens. |
| --- |


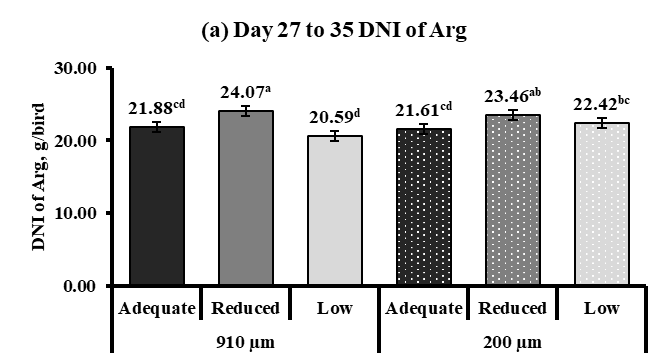

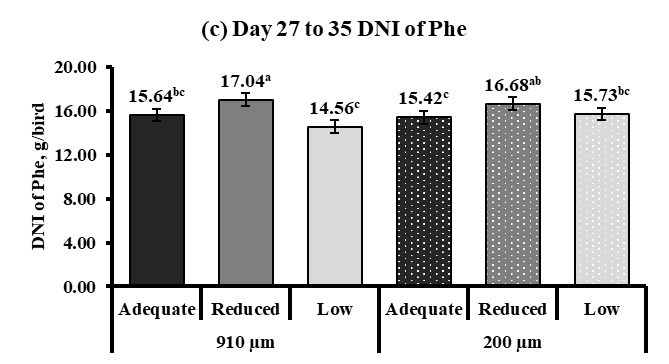

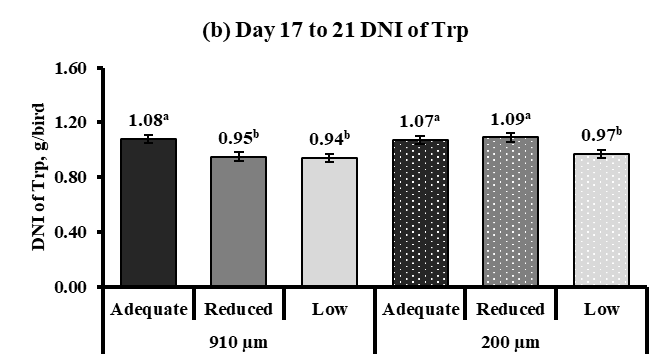

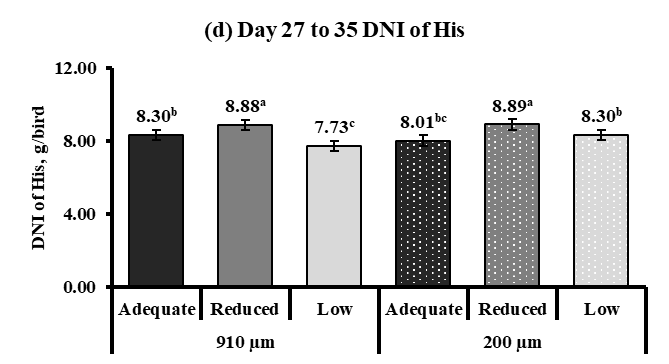


| **Figure S5**. Interaction effects^1^ of varying limestone particle size (910 and 200 µm) and Ca concentrations (adequate, reduced, and low) fed to YPM x Ross 708 male broilers on (**a**) d 27 to 35 DNI of Arg (*P* = 0.001; CLM ± 0.69), (**b**) d 17 to 21 DNI of Trp (*P* < 0.001; CLM ± 0.03), (**c**) d 27 to 35 DNI of Phe (*P* = 0.016; CLM ± 0.59), and (**d**) d 27 to 35 DNI of His (*P* = 0.007; CLM ± 0.28). ^a-d^Means with different superscripts differ significantly (*P* ≤ 0.05). ^1^Interaction values are least square means of 10 replicate pens. |
| --- |


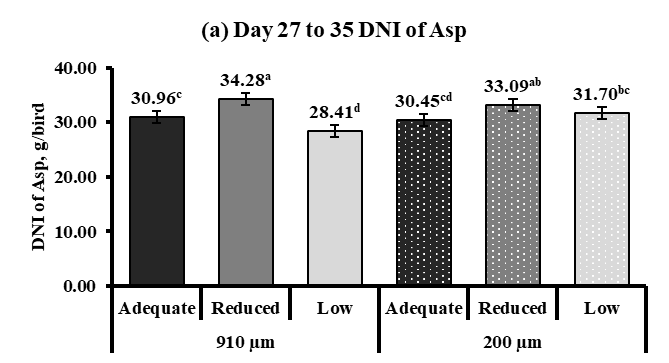

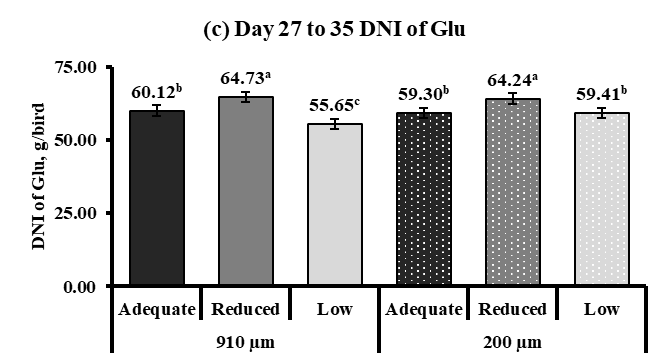

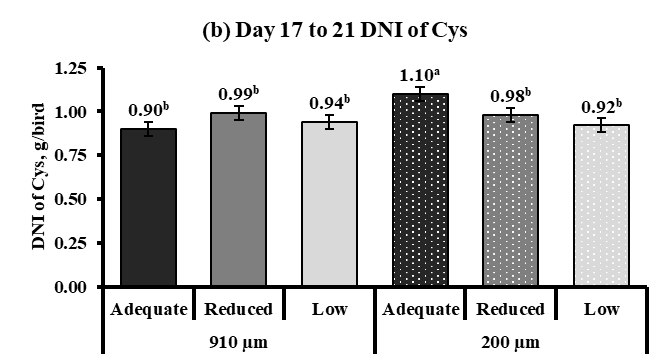

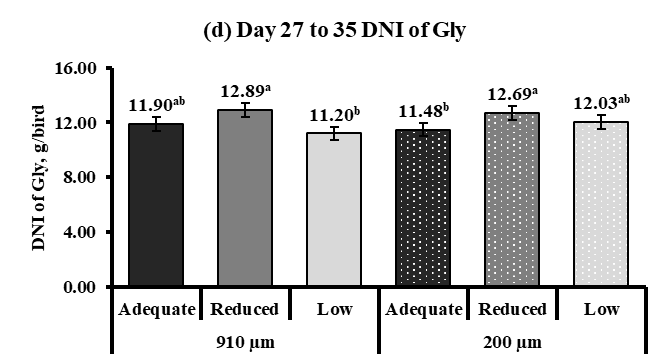


| **Figure S6**. Interaction effects^1^ of varying limestone particle size (910 and 200 µm) and Ca concentrations (adequate, reduced, and low) fed to YPM x Ross 708 male broilers on (**a**) d 27 to 35 DNI of Asp (*P* < 0.001; CLM ± 1.09), (**b**) d 17 to 21 DNI of Cys (*P* < 0.001; CLM ± 0.04), (**c**) d 27 to 35 DNI of Glu (*P* = 0.011; CLM ± 1.79), and (**d**) d 27 to 35 DNI of Gly (*P* = 0.028; CLM ± 0.50). ^a-d^Means with different superscripts differ significantly (*P* ≤ 0.05). ^1^Interaction values are least square means of 10 replicate pens. |
| --- |


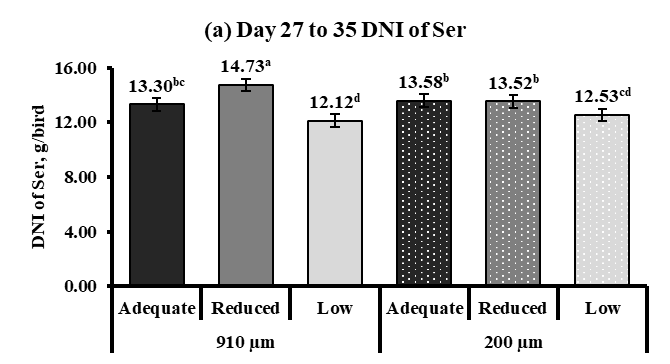

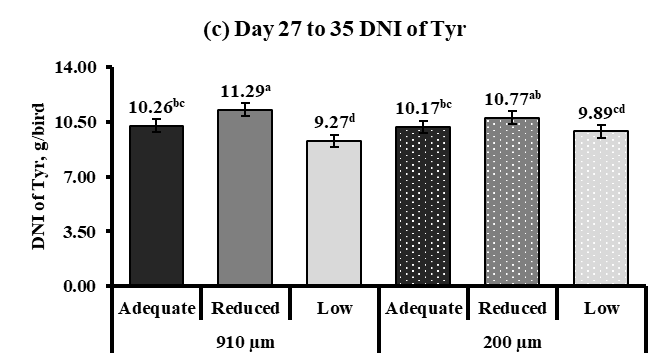

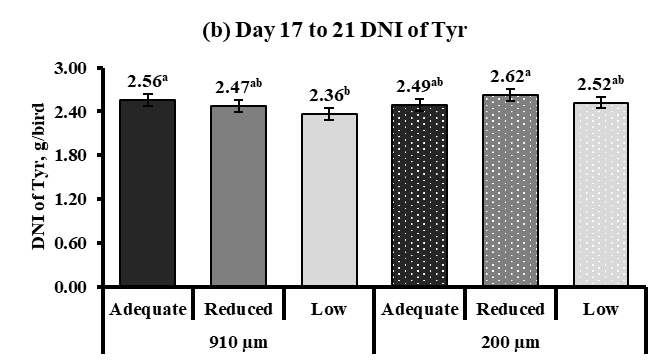


| **Figure S7**. Interaction effects^1^ of varying limestone particle size (910 and 200 µm) and Ca concentrations (adequate, reduced, and low) fed to YPM x Ross 708 male broilers on (**a**) d 27 to 35 DNI of Ser (*P* = 0.001; CLM ± 0.45), (**b**) d 17 to 21 DNI of Tyr (*P* = 0.010; CLM ± 0.08), and (**c**) d 27 to 35 DNI of Tyr (*P* = 0.019; CLM ± 0.40). ^a-d^Means with different superscripts differ significantly (*P* ≤ 0.05). ^1^Interaction values are least square means of 10 replicate pens. |
| --- |
